# Novel *GATA1* Variant Causing a Bleeding Phenotype Associated with Combined Platelet α-/δ-Storage Pool Deficiency and Mild Dyserythropoiesis Modified by a *SLC4A1* Variant

**DOI:** 10.3390/cells11193071

**Published:** 2022-09-29

**Authors:** Kerstin Jurk, Anke Adenaeuer, Stefanie Sollfrank, Kathrin Groß, Friederike Häuser, Andreas Czwalinna, Josef Erkel, Nele Fritsch, Dana Marandiuc, Martin Schaller, Karl J. Lackner, Heidi Rossmann, Frauke Bergmann

**Affiliations:** 1 Center for Thrombosis and Hemostasis (CTH), University Medical Center of the Johannes Gutenberg University, 55131 Mainz, Germany; 2 Institute for Clinical Chemistry and Laboratory Medicine, University Medical Center of the Johannes Gutenberg University, 55131 Mainz, Germany; 3 Coagulation Laboratory, MVZ Wagnerstibbe, Amedes-Group, 30159 Hanover, Germany; 4 Pediatric Department, St. Marienhospital, 49377 Vechta, Germany; 5 Transfusion Center, University Medical Center of the Johannes Gutenberg University, 55131 Mainz, Germany; 6 Department of Dermatology, University of Tübingen, 72076 Tübingen, Germany

**Keywords:** inherited platelet disorders, storage pool deficiency, thrombocytopenia, anemia, *GATA1*, *SLC4A1*, whole exome sequencing

## Abstract

Germline defects in the transcription factor GATA1 are known to cause dyserythropoiesis with(out) anemia and variable abnormalities in platelet count and function. However, damaging variants closely located to the C-terminal zinc finger domain of GATA1 are nearly unknown. In this study, a 36-year-old male index patient and his 4-year-old daughter suffered from moderate mucocutaneous bleeding diathesis since birth. Whole exome sequencing detected a novel hemizygous *GATA1* missense variant, c.886A>C p.T296P, located between the C-terminal zinc finger and the nuclear localization sequence with non-random X-chromosome inactivation in the heterozygous daughter. Blood smears from both patients demonstrated large platelet fractions and moderate thrombocytopenia in the index. Flow cytometry and electron microscopy analysis supported a combined α-/δ (AN-subtype)-storage pool deficiency as cause for impaired agonist-induced platelet aggregation (light transmission aggregometry) and granule exocytosis (flow cytometry). The absence of BCAM in the index (Lu(a-b-)) and its low expression in the daughter (Lu(a-b+)) confirmed a less obvious effect of defective GATA1 also on erythrocytes. Borderline anemia, elevated HbF levels, and differential transcription of GATA1-regulated genes indicated mild dyserythropoiesis in both patients. Furthermore, a mild SLC4A1 defect associated with a heterozygous *SLC4A1* c.2210C>T p.A737V variant maternally transmitted in the daughter may modify the disease to mild spherocytosis and hemolysis.

## 1. Introduction

Platelets, the smallest blood cells, crucially control primary and secondary hemostasis and modulate intravascular processes involved in inflammation and immunity [1,2]. Megakaryocytes, platelet precursors localized in the bone marrow and lungs, release about 10^11^ platelets per day yielding in a platelet count of 150–450 × 10^9^/L with a platelet life span of about 10 days [3].

Inherited platelet disorders represent a group of more than 50 rare diseases characterized by reduced platelet number (thrombocytopenia) and/or platelet function defects affecting platelet adhesion, granule secretion, thromboxane A2-release, aggregation, and coagulant activity. Defects in genes responsible for megakaryopoiesis and platelet production are well known to cause inherited low platelet count less than 150 × 10^9^/L and frequently enlarged platelets (macrothrombocytopenia). Bleeding diathesis is typically characterized by mucocutaneous bleeding symptoms (e.g., petechiae, ecchymoses, epistaxis, menorrhagia), and their risk increases for severe isolated thrombocytopenias (<50 × 10^9^ platelets/L). Interestingly, the bleeding severity varies from nearly absent to life-threatening episodes, which is not exclusively explained by the degree of platelet count reduction [4,5,6]. A recent study by the Scientific and Standardization Committee (SSC) “Platelet Physiology” of the International Society on Thrombosis and Haemostasis (ISTH) evaluated the ISTH bleeding assessment tool (ISTH-BAT) for platelet function and platelet (low) number disorders on the basis of more than 1000 patients from 17 countries worldwide [7]. This validation study demonstrated that the discrimination power compared to healthy controls was excellent for platelet function disorders (e.g., Glanzmann thrombasthenia, δ-storage pool disorder, primary secretion defects) but poor for thrombocytopenias (e.g., MYH9-related disorders, monoallelic Bernard Soulier syndrome, ANKRD26- and ETV6-related thrombocytopenias), which were also associated with a lower frequency of (clinically relevant) bleeding symptoms.

Several transcription factors are importantly involved in the regulation of megakaryopoieses and platelet formation. Pathogenic germline variants in genes encoding the prominent transcription factors RUNX1, GATA1, FLI1, GFI1b, and ETV6 are known to cause thrombocytopenias, associated with large and normal platelet size, respectively [8,9]. The GATA binding protein GATA1 is one of the best studied transcription factors, whose germline defects lead to dyserythropoiesis with or without anemia and variable platelet abnormalities. GATA1 is encoded by six exons located on the X-chromosome and comprises 413 amino acids including 2 conserved zinc finger domains.

Most of the germline defects in GATA1 characterized by X-linked inheritance were published to be caused by pathogenic missense variants located within the N-terminal zinc finger domain. These mutations either prevent the interaction of GATA1 with important regulatory co-factors such as friend of GATA1 (FOG1), or destabilize the binding of non-canonical and palindromic GATA1 motifs to DNA. Decreased affinity of GATA1 for FOG1 has been shown to be caused by the pathogenic missense variants V205M, G208S, and D218G/Y, which are associated with variable dyserythropoiesis (with or without anemia), dysplastic megakaryocytes, and macrothrombocytopenia (OMIM: 300367; 300835). These defects can be associated with reduced expression of megakaryocyte-specific genes encoding GPVI, platelet factor 4, and proteins of the GPIb-V-IX von Willebrand receptor complex. Originally described as X-linked thrombocytopenia with beta-thalassemia (OMIM: 314050), the N-terminal zinc finger domain variants R216Q/W have been shown to affect GATA1 DNA binding sites but not interaction with FOG1 [9,10,11].

As paucity or absence of platelet α-granules is another typical hallmark of GATA-related thrombocytopenias, dysfunctional platelets show distinct similarities, with the gray platelet syndrome, which is caused by mutations in *NBEAL2* encoding neurobeachin 2, containing Beige and Chediak Higashi (BEACH) domains [12,13].

However, damaging variants located within or near the C-terminal zinc finger domain of GATA1 and their effects on platelets and erythropoiesis are incompletely understood. In this study, we describe a male patient with novel hemizygous GATA1 variant T296P located between the C-terminal zinc finger (aa258–282, Uniprot) and the nuclear localization sequence (aa301–319 [14]), as well as his affected and unaffected family members. We hypothesized an association between this novel C-terminal GATA1 variant and defects of platelets and erythrocytes in the affected patients with bleeding diathesis.

## 2. Materials and Methods

### 2.1. Index Patient and Family Members

**Figure 1:**
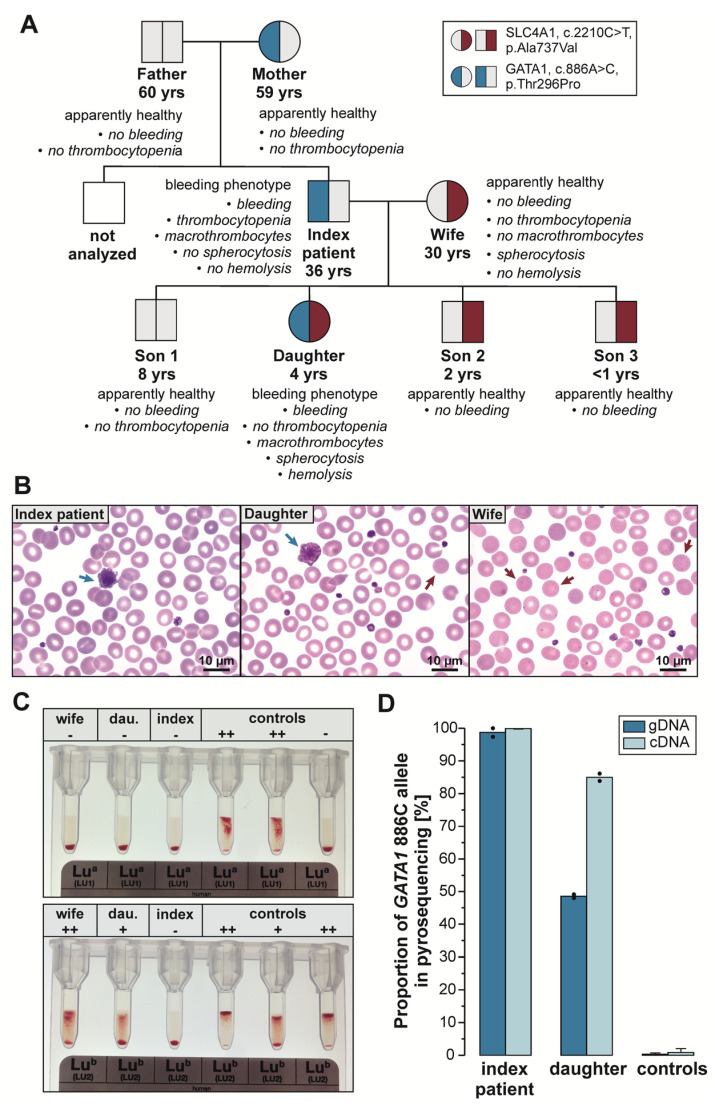
(**A**) Family pedigree with genotype for *GATA1*: c.886A>C p.(Thr296Pro) and *SLC4A1*: c.220C>T p.(Ala737Val), age at diagnosis, and individual phenotype. (**B**) Blood smear of index patient (left), his daughter (middle), and his wife (right), showing large platelets (blue arrows) and microspherocytes (exemplary, red arrows). Magnification: 100× (oil). (**C**) Gel column agglutination assay for Lutheran blood group typing. All family members (index case, daughter (dau.), and wife) were Lu^a^ negative (upper panel). The wife showed the highest amount of detectable Lu^b^ (reaction strength 2+), the daughter was Lu^b^ positive but weaker (reaction strength 1+), whereas the index patient was Lu^b^ negative. (**D**) Analysis of X-chromosome inactivation via allele quantification of cDNA and gDNA using pyrosequencing and the pathogenic allele 886C (*GATA1*). The index patient’s daughter showed a higher cDNA amount of the pathogenic variant than expected, revealing an imbalanced X-inactivation (≈86% variant allele).

Affected members of the described Caucasian family presented with moderate bleeding diathesis. The affected 36-year-old male index patient suffered from frequent and severe epistaxis and hematoma since birth (ISTH-BAT score: 9). He never underwent elective surgery. The affected 4-year-old daughter suffered from multiple light red hematomas after even minimal trauma (ISTH-BAT score: 5). Von Willebrand disease and sub-hemophilia could be excluded for both patients. The index patient’s parents and three sons as well as his wife (30 years old) did not show any bleeding symptoms (Figure 1A).

### 2.2. Blood Count, Clinical Chemistry, and HPLC

Family members were subjected to a set of routine blood analyses. Clinical chemistry analyses (C-reactive protein (CRP), ferritin, transferrin, transferrin saturation, and haptoglobin) were performed on an Architect c8000 system (Abbott, Wiesbaden, Germany). Erythropoietin was measured on an Immulite 2000 (Siemens Healthcare Diagnostics, Eschborn, Germany). Whole blood counts were recorded on an Advia 2120i Hematology System (Siemens Healthcare GmbH, Erlangen, Germany). Hb-electrophoresis was performed on a Variant II Hemoglobin Testing System (BioRad, Feldkirchen, Germany). Blood smear was prepared and stained (Wright-Giemsa) using the Advia 2120i and analyzed with a Leica DM200 microscope with a Leica DFC295 camera (Leica Biosystems, Nussloch, Germany). All tests were carried out with the reagents and according to the instructions of the respective device manufacturers.

### 2.3. Whole Exome Sequencing

Genomic DNA (gDNA) was isolated from 200 µL EDTA whole blood with the QIAamp DNA Mini Kit (Qiagen, Hilden, Germany). Library preparation was performed for the index patient and his wife, daughter, and the oldest son (son 1) using the xGen Exome Research Panel (version 1, Integrated DNA Technologies, Coraville, IA, USA). Sequencing was performed externally (300 bp paired read; HiSeq; Illumina, San Diego, CA, USA). Alignment and variant calling were conducted using NextGENe (Softgenetics, State College, PA, USA). An in-house Perl script was subsequently applied for gene and variant filtering. The genes filtered belong to a coagulation disorder panel (322 genes), an anemia panel (96 genes), and the genes found to be deregulated in the index patient’s platelet proteome analysis (46 genes, Supplementary Methods). *BCAM* and *KLF1* were added due to the results of the Lutheran antigen analysis, resulting in 495 genes analyzed in total (exclusion of duplicates).

To confirm the next-generation sequencing (NGS) results and to screen further family members for the respective variants, Sanger sequencing (Beckman CEQ 8000 Genetic Analysis System, Sciex, Darmstadt, Germany) and pyrosequencing (PyroMark Q96 ID, Qiagen, Hilden, Germany) were used and performed as described earlier [15,16]. Primers and conditions used are listed in Tables S1 and S2.

### 2.4. X-Chromosome Inactivation

X-chromosome inactivation was studied in the index patient, his wife, his daughter, and two controls considering *GATA1:* c.886A>C p.(Thr296Pro) in gDNA and cDNA by allele quantification via pyrosequencing. The primers and conditions applied are listed in Table S3. Total RNA was extracted using the PAXgene Blood RNA-System (PreAnalytiX, Qiagen, Hilden, Germany). First-strand cDNA was synthesized according to the manufacturer’s instructions by SuperScript III reverse transcriptase (Invitrogen, Thermo Fisher Scientific, Waltham, MA, USA) with Oligo(dT)_12–18_ primers (Invitrogen, Thermo Fisher Scientific, Waltham, MA, USA). Two further X-chromosomal markers (*ELF4* (rs2181440, minor allele frequency (MAF) (T): ≈25%); *PRPS2* (rs1731469, MAF (A): ≈62%) were analyzed for confirmation. A calibration factor was calculated for each assay by correcting the values of genomically homozygous carriers and non-carriers to 100% and 0%, respectively. Calibrated values <0% or >100% were cut to represent 0–100%.

### 2.5. Lutheran Blood Group Typing

The Lu^a^ and Lu^b^ antigens of the Lutheran blood group system were determined with a gel column agglutination assay on ID-cards containing polyclonal antibodies (ID-Card “Anti-Lua”, ID-Card “Anti-Lub”, BioRad, Cressier, Switzerland). According to the manufacturer’s instructions, 5% erythrocyte suspension was added to a microtube and centrifuged for 10 min.

The Lutheran antigens were determined to check for *BCAM* protein expression in the index patient, his wife, and his daughter.

### 2.6. Preparation of Platelet-Rich and Platelet-Free Plasma

Citrate-anticoagulated (3.2% (*v*/*v*) tri-sodium citrate) whole blood was collected in a standardized manner and processed within one hour after blood collection to prevent artificial platelet activation ex vivo [17]. Centrifugation at 200× *g* for 10 min at room temperature yielded in a supernatant of platelet-rich plasma. The remaining buffy-coat was centrifuged at 2000× *g* at room temperature to prepare a supernatant of platelet-poor plasma [18].

### 2.7. Preparation of Washed Platelets for Electron Microscopy

Washed human platelets were prepared as described [19]. The isolated platelets were adjusted to 5 × 10^8^/mL and kept resting at 37 °C for 15 min.

### 2.8. Preparation of Gel-Filtered Platelets for Immunoblot Analysis and ELISA Assays

Gel-filtered human platelets were prepared from platelet-rich plasma as described [17]. The isolated platelets were diluted 1:1 with platelet washing buffer (120 mM NaCl, 12.9 mM citrate, 30 mM glucose; pH 6.5) and centrifuged at 400× *g* for 10 min at room temperature. Pelleted platelets were adjusted to 2 × 10^8^/mL with Tyrode’s buffer, pH7.4, and lysed by freezing at −80 °C.

### 2.9. Light Transmission Aggregometry

Agonist-induced platelet aggregation according to Born [20] was measured as described previously with some modifications [21]. The platelet count in platelet-rich plasma was adjusted to 2 × 10^8^/mL with autologous platelet-poor plasma to compare platelet aggregation responses with lumi-aggregometry analysis (Section 2.10). ADP (5 µM, Sigma-Aldrich, St. Louis, MO, USA), the thrombin receptor PAR-1-activating peptide TRAP-6 (20 µM, Bachem Biochemica GmbH, Heidelberg, Germany), epinephrine (2 µM, Sigma-Aldrich), and the thromboxane A_2_ (TxA_2_) mimetic U46619 (3 µM, Sigma-Aldrich) were used as platelet agonists according to the recommendation of the SSC “Platelet Physiology” of the ISTH [22].

### 2.10. Lumi-Aggregometry

Simultaneous monitoring of agonist-induced platelet aggregation by light transmission aggregometry and ATP release from δ-granules was performed in a Chronolog-lumi-aggregometer (Chrono-log model 700, Harvertown, PA, USA) according to the manufacturer’s instructions. The platelet count in platelet-rich plasma was adjusted to 2 × 10^8^/mL with platelet-poor plasma. Platelet ATP release was detected and quantified by the luciferase-catalyzed conversion of luciferin, leading to generation of bioluminescence in the presence of ATP [23]. Equine collagen type I (1 or 10 µg/mL, Chrono-Par, Harvertown, PA, USA) and arachidonic acid (0.5 mg/mL, Sigma-Aldrich) were used as platelet agonists according to the recommendation of the SSC “Platelet Physiology” of the ISTH [22].

### 2.11. Flow Cytometric Analysis of Platelet Receptor Surface Expression

Diluted citrated whole blood (1:1 with PBS, pH 7.4) was incubated with saturating concentrations of mouse IgG1-FITC, anti-human CD41/CD61-FITC (clone P2, Beckman Coulter, Krefeld, Germany), anti-human CD42b-FITC (clone SZ2, Beckman Coulter), anti-human CD42a (clone Beb1, Becton Dickinson, Heidelberg, Germany), anti-human CD29-FITC (clone K20, Beckman Coulter), and anti-human GPVI-eFluor660 (clone HY101, eBioscience, Frankfurt, Germany) antibodies for 20 min at room temperature. The samples were further diluted 1:100 with PBS, pH 7.4, and 10.000 platelets, which were identified by FSC/SSC properties, and were measured using a FACSCanto II flow cytometer (Becton Dickinson (BD) Biosciences, Heidelberg, Germany). Quantum™ Simply Cellular^®^ anti-mouse IgG kit (Bangs-Laboratories, Fishers, IN, USA) was used to quantify absolute numbers of antigen binding sites (ABS) per platelet according to the manufacturer’s instructions [24]. FACS Diva software (v6.1.3 BD Biosciences, Heidelberg, Germany) was used for data analysis.

### 2.12. Flow Cytometric Analysis of Platelet Functions

Diluted platelet-rich plasma (1:10 with Tyrode’s buffer) was treated with increasing concentrations of the collagen receptor GPVI selective agonist convulxin (DSM, Nutritional Products Ltd., Branch Pentapharm, 4147 Aesch BL, Switzerland) for 6 min at room temperature and fixed with 0.5% (*v*/*v*) formaldehyde (final concentration) for 30 min at room temperature. Platelets were washed once with Tyrode’s buffer and subsequently centrifuged at 800× *g* for 10 min at room temperature. Pelleted platelets were incubated with anti-human CD62P-FITC (clone AK-4, 5 µg/mL, BD Biosciences) and anti-human CD63-FITC (clone H5C6, 5 µg/mL, BD Biosciences) antibodies for 30 min at room temperature. Platelets were analyzed by flow cytometry as described in Section 2.11. Data were presented as linear values of the mean fluorescence intensity (MFI).

### 2.13. Transmission Electron Microscopy

Washed platelets were fixed for 10 min at 37 °C using Karnovsky’s fixative (3% formaldehyde, 2.5% glutaraldehyde, 0.1 M phosphate buffer; pH 7.4) and stored at 4 °C until further processing. Pelleted platelets were embedded in 3.5% agarose at 37 °C and fixed again in Karnovsky’s fixative. Post-fixation was performed on 1.0% osmium tetroxide containing 1.5% K-ferrocyanide in 0.1 M cacodylate buffer for 2 h. Agarose blocks were embedded in glycide ether. Ultra-thin sections (30 nm, ultramicrotome, Ultracut, Reichert, Vienna Austria) were mounted on copper grids and analyzed using a transmission electron microscope operating at 120 kV (Zeiss LIBRA 120, Carl Zeiss, Oberkochen, Germany).

### 2.14. Immunoblot Analysis

Analysis of Western blot samples was performed with washed platelets as described [19]. Proteins were separated by SDS-PAGE (8%) and transferred to polyvinylidene difluoride (PVDF) membranes. Monoclonal anti-human CD42b (clone SZ2, 1:200, Beckman Coulter), anti-human CD62P/P-selectin (clone AK-4, 1:40, non-reduced conditions, BD Biosciences), and anti-human CD63 (clone H5C6, 1:40, non-reduced conditions, BD Biosciences) antibodies as well as polyclonal rabbit anti-human β-actin antibodies (1:3000, Cell Signaling Technology, MA, USA) were used as primary antibodies. Polyclonal goat anti-mouse or goat anti-rabbit IgG conjugated with horseradish peroxidase was used as the secondary antibodies (1:5000). Membranes were developed by enhanced chemiluminescence (ECL) detection.

### 2.15. Quantification of Serotonin of Gel-Filtered Platelets

Serotonin was quantified in lysates of gel-filtered platelets using an ELISA kit (IBL International RE59121, Hamburg, Germany) according to the manufacturers’ protocols.

### 2.16. mRNA Profiling

mRNA was isolated from whole blood using the PAXgene Blood RNA-System (PreAnalytiX, Qiagen, Hilden, Germany). The transcript profiling was performed on a NextSeq500 instrument using the NextSeq 500/550 High Output Kit v2.5 (75 Cycles) (Illumina, San Diego, CA, USA), as well as the TruSeq Stranded mRNA Library Prep Kit (Illumina, San Diego, CA, USA) for library preparation. Transcriptome assembly and differential expression analysis were performed on Galaxy Europe [25] (among others, STAR and Cuffdiff). Transcription data from the index patient, his wife, and his daughter were compared to a healthy control and filtered for the genes deregulated in the index patient’s platelet proteome and 49 genes known to be regulated by GATA1 (Supplementary Methods). Significance calculations were adjusted for multiple testing, and a q value of <0.05 was considered significant.

### 2.17. Transcript Analysis via Digital Droplet PCR (ddPCR)

Singleplex ddPCR assays were established to confirm several mRNA profiling results (*SLC4A1, ANK1, TMCC2, KLF1, AHSP, GATA1, CXCL12, DOCK10, ENO2, LGALS1*) and to analyze transcripts that could not be evaluated using the NGS approach (*HGB1/2, HBA1/2,* and *HBB*). cDNA acquisition was performed as described in Section 2.4. The QX200™ system (BioRad, Feldkirchen, Germany) and EvaGreen chemistry (QX200 ddPCR EvaGreen^®^ Supermix, BioRad, Feldkirchen, Germany) were used according to the manufacturer’s instructions. Primers were designed for an annealing temperature of 56 °C and are listed in Table S4. cDNA copies per µL were calculated by the QuantaSoft™ Analysis Pro (1.0.596) (BioRad, Feldkirchen, Germany) software. The transcripts of the index patient, his wife and daughter, and three healthy controls were quantified in relation to the expression of a control gene (*SDHB, MRPL9*, or *ACTB)*. A fold change was calculated by dividing the relative transcription level of target genes of the subjects by the averaged transcription level of healthy controls. The significance of expression differences was determined using a T-test for independent samples. A *p*-value of <0.05 was considered statistically significant. The data were analyzed using Origin 7.5 V5 software (OriginLab Corporation, Northampton, MA, USA).

## 3. Results

### 3.1. Complete Blood Count, and Platelet and Erythrocyte Characteristics

The white blood cell count, including the automated differential count, was normal for the index patient, his affected daughter, and his unaffected wife. This was in line with normal C-reactive protein values (Table 1).

In contrast to his daughter and wife, the index patient was characterized by mild thrombocytopenia (88 × 10^3^/µL). Although the mean platelet volume was within the normal range (Table 1), both the index patient’s and his daughter’s blood smears demonstrated the presence of large platelets/macrothrombocytes (Figure 1B). In comparison, the index patient’s wife did not show an abnormal platelet count or phenotype.

Mild changes in erythropoiesis were observed in the index patient and his daughter (Table 1). Both presented with borderline anemia (hemoglobin: index: 12.5 g/dL, ref. >13.5 g/dL); daughter: 10.9 g/dL, ref. >10 g/dL), elevated levels of fetal hemoglobin (HbF, index: 2.8%; daughter: 13.5%, ref. <1%), and elevated reticulocyte count (index: 210/nl; daughter: 114/nl, ref. <69/nl). As observed in the index patient’s wife and, in contrary to the index, spherocytes were detected in the daughter’s blood smear. While mean corpuscular volume (MCV, 100.3 fl, reference range (ref.) 83–100 fl) and mean corpuscular hemoglobin (MCH, 33.7 pg, ref. 27–33 pg) were slightly above the upper reference range in the index patient (mean corpuscular hemoglobin concentration (MCHC) 31.9 g/dL, ref. 32–35 g/dL), the daughter showed a MCHC (34.3 g/dL) at the upper limit of the reference range in combination with comparatively low MCV (87.6 fl) and MCH (30.0 pg). A similar but even more pronounced erythrocyte pattern was detected in the index patient’s wife (MCV 82.3 fl, MCH 28.5 pg, MCHC 34.6 g/dL). Like her husband and daughter, she had mild reticulocytosis (78/nL). Iron deficiency was absent in the index (ZPP 75 mmol/mol heme; iron deficiency: >100 mmol/mol heme) and the daughter (ZPP 80 mmol/mol heme) and at most mild in the wife (ZPP 104 mmol/mol heme). Therefore, the family’s pattern of red blood cell indices and morphology suggested two genetic causes. One affected the index, the other affected his wife, and both were inherited to the daughter. This notion is supported by the fact that although the daughter’s bleeding symptoms appear to be less severe, her red cell lineage seems more severely affected than that of her father and resembles aspects observed in her mother. The daughter was the only one showing hemolysis (haptoglobin undetectable) and an elevated erythropoietin concentration (37.1 mU/mL (ref. <29)), and both reticulocyte and HbF increase were more prominent than in her parents. The blood count of the index’s father, mother, and son 1 was largely unremarkable (Table S5). No fresh EDTA blood samples were available from the other family members.

### 3.2. Molecular Genetic Testing

Exome sequencing was carried out on the index, his wife, and two of their children (daughter, son 1). Synonymous or common variants (MAF > 1%) were sorted out. Relevant variants were selected by a candidate gene approach using two filter lists combined with pedigree information (Table 2). Filtering by a list of 322 coagulation genes (Supplementary Methods), taking into account the pedigree information about bleeding symptoms, revealed three variants: *GATA1*: c.886A>C p.(Thr296Pro), *EPHB1*: c.1856T>C p.(Val619Ala), and *AP3B1*: c.1069A>G p.(Ile357Val). They were found hemizygous (*GATA1*) or heterozygous (*EPHB1, AP3B1*) in the index, and heterozygous in the affected daughter, but were absent from the wife and the apparently healthy son 1 (Figure S1). Filtering by a further anemia targeting genes list (96 genes; Supplementary Methods) and taking into account the blood count/smear abnormalities, indicating mild spherocytosis (not available from sons 1–3), resulted in a *SLC4A1* candidate variant (c.2210C>T p.(Ala737Val)), which was found heterozygous in the affected wife and daughter, but was absent in the index and son 1 (Figure S2). Either pyrosequencing or Sanger sequencing confirmed the presence of these four interesting variants and were applied to genotype further family members (EDTA blood from father, mother, and son 2, and a buccal swab from son 3; Figures S1 and S2).

### 3.3. Classification of the Candidate Variants Potentially Causing a Platelet Disorder

The fact that neither parent of the index patient suffered from a bleeding disorder suggested autosomal or X-linked recessive inheritance, a criterion that was only met by the *GATA1* variant c.886A>C p.(Thr296Pro). The *GATA1* gene is located on the X-chromosome, and the index is hemizygous for the variant. Initially, the affected daughter seemed to contradict the suspected mode of inheritance, but pyrosequencing-based allele quantification for *GATA1* 886C (variant allele, Figure 1D) and 886A, as well as the analysis of two further X-linked markers (*ELF4* (rs2181440); *PRPS2* (rs1731469), data not shown) from the index patient’s and his daughter’s gDNA and cDNA revealed non-random X-inactivation in the daughter. Although heterozygous, only ≈10% less variant allele expression was observed in her than in the index (index: 100% gDNA, ≈100% cDNA; daughter: 50% gDNA, ≈86–93% cDNA). From the mother of the index, who also carries the *GATA1* variant, no material for RNA isolation was available, hindering the investigation of her X-inactivation. Since she is not affected by a bleeding disorder, we assumed a random X-inactivation, and thus only ≈50% varied GATA1 in the mother.

GATA1 belongs to the family of GATA-motif-binding transcription factors. It is a key regulator of hematopoiesis and involved in the fetal development and adulthood maturation of the three lineages, namely, erythropoiesis, thrombopoiesis, and leukopoiesis [10,26,27]. Depending on the domain in which the defect is located and whether it occurred somatically or in the germline, GATA1 variants cause very different symptoms of varying severity. Germline variants, which are not associated with an N-terminally truncated protein, cause (macro)thrombocytopenia with varying changes in the red cell lineage, ranging from no alteration (XLT, OMIM 300367) to dyserythropoietic anemia (XLTDA, OMIM 300367) or severe β-thalassemia-like anemia (XLTT, OMIM 314050). Thus, the symptoms of the index (macrothrombocytopenia, platelet dysfunction, and mild dyserythropoiesis) and his daughter (similar symptoms, but normal platelet count) are in good agreement with a GATA1 defect in the C-terminal part of the protein. T296P is located between the C-terminal zinc finger (aa258–282, Uniprot) and the nuclear localization sequence (aa301–319 [14]). In this region (aa283–300), no common variants (MAF > 1%) and only two pathogenic variants affecting the same amino acid (H289Y/T) have been reported [28,29]. T296 is highly conserved among species (PolyPhen2) and among members of the GATA family expressed in the bone marrow and peripheral blood cells (GATA1-3) [26]. The exchange of threonine by proline is non-conservative with an impact on protein structure since threonine is a polar amino acid and proline a non-polar, cyclic amino acid. Bioinformatics tools consistently predict pathogenicity. To prove a functional effect of GATA1 T296P, we analyzed the Lu^a^ and Lu^b^ antigens since a Lu(a-b-) phenotype has already been described for a C-terminal variant of *GATA1* [30]. The absence of these antigens, which are part of the Lutheran blood group system [31], is rare in Caucasians (e.g., 1:3000–5000 in Great Britain [32]) and indicates the absence or very low expression of BCAM (basal cell adhesion molecule). Apart from variants in the BCAM gene, the Lu(a-b-) phenotype is caused by variants in *KLF1* [33]. KLF1 is a key regulator of erythropoiesis and closely cooperates with GATA1 [34]. As no pathogenic variant was detected in *BCAM* and *KLF1*, and since *BCAM* expression was absent in the father (Lu(a-b-), Figure 1C) and low in the daughter (Lu(a-b+) with low strength Lu^b^), a functional defect of GATA1 through T296P could be proven in both patients.

In addition to the *GATA1* variant, two other candidate variants were defined (*AP3B1*: c.1069A>G p.(Ile357Val); *EPHB1*: c.1856T>C p.(Val619Ala)), which, however, are less likely to cause bleeding symptoms. Since both are present a in heterozygous state in the index and his daughter, and since no second variant was found in the same gene, the resulting genotypes do not match the expected recessive inheritance. Variants in *AP3B1* are a known cause of the Hermansky–Pudlak syndrome 2, a rare autosomal recessive disease characterized by platelet defects and oculocutaneous albinism (OMIM 608233). Furthermore, bioinformatics predictions are inconsistent. Since neither the phenotype nor the mode of inheritance or the frequency of the candidate variant *AP3B1* Ile357Val (MAF of 0.1%) matches the family disease description, this variant could be ruled out. EPHB1 is expressed in platelets and was suggested to be involved in clot stability [35], but it has never been linked to a bleeding disorder. EPHB1 Val619Ala is rare, and bioinformatics consistently predicts its pathogenicity, but its suggested mode of inheritance and function are not in accordance with the family’s phenotype. Since we found each of the two variants also in healthy family members (*AP3B1*: father, son 2; *EPHB1*: mother) and since we could prove the functionality of GATA1 T296P, we assessed the variants as likely benign (*AP3B1*) and a variant of uncertain significance (VUS; *EPHB1*), even though they cannot be ruled out as potential modifiers of the disease.

### 3.4. Classification of the SLC4A1 Variant Causitive for Mild Spherocytosis

Evidence for mild spherocytosis in the index’s wife and daughter prompted the evaluation of the exome sequencing results with respect to anemia-causing genes. Candidate variant c.2210C>T p.(Ala737Val) in *SLC4A1* was found in a heterozygous state in both, while it was absent from the non-affected family members (index, son 1, mother, and father). *SLC4A1*-induced spherocytosis usually shows dominant inheritance (Table 2), which fits with the transmission in the family. The variant was also detected in sons 2 and 3, but neither blood count nor blood smear were available, hindering the detection of signs of mild spherocytosis. Only the hemoglobin HPLC from frozen EDTA blood of son 2 showed an elevated HbF (5.5, ref. <1), possibly indicating an increased hematopoiesis. As elevated HbF levels were also found in the index (2.8%), his wife (1.6%), and his daughter (13.5%), known causative variants in the coding sequence, splice sites, and promoter regions of the *HBA* and *HBB* gene cluster were excluded as causes by analysis of the exome data. The amino acid exchange alanine to valine at position 737 in SLC4A1 is conservative and located in the C-terminal, cytosolic side of transmembrane domain 10 (aa720–737, NM_000342.4). Variants causing hereditary spherocytosis are often located close to the cytosolic end of a transmembrane helical segment [36]. These substitutions are prone to destabilize the helix or to interfere with helix–helix interactions. In accordance, bioinformatics tools consistently predict pathogenicity. Ala737Val is rare but listed in dbSNP and ClinVar (VUS). According to ACMG criteria and with respect to the mRNA expression analyses detailed in Section 3.8, we classified *SLC4A1*: c.2210C>T p.(Ala737Val) as pathogenic. Elevated HbF is a common finding in increased erythropoiesis as it occurs in spherocytosis and GATA1-mediated dyserythropoiesis. Therefore, the presence of SLC4A1 A737V in the daughter may modify the GATA1-mediated disease, possibly explaining her high HbF levels and hemolysis.

### 3.5. Platelets from Index Patient and His Daughter Showed Impaired Aggregation in Response to ADP, Epinephrine, TRAP-6, and TxA2 Mimetic U46619, but Normal Surface Expression of Major Receptors

**Figure 2:**
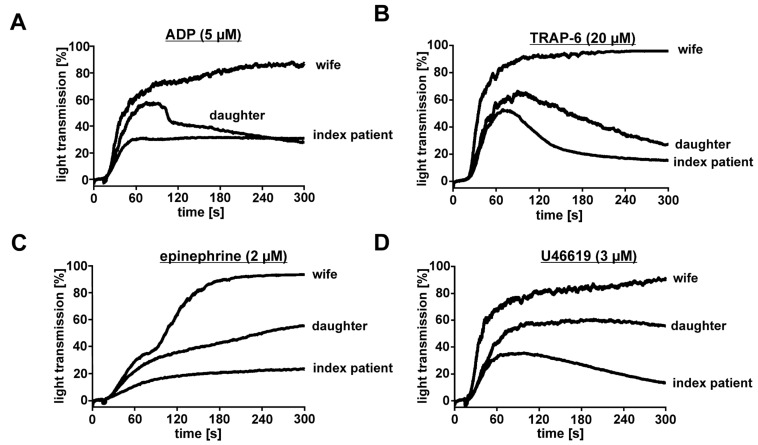
Agonist-induced aggregation of platelets from the GATA1-T296P-variant-affected index patient, his daughter, and his unaffected wife. Platelet aggregation was determined by light transmission aggregometry in platelet-rich plasma with a platelet concentration of 2 × 10^8^/mL adjusted with autologous platelet-poor plasma. Platelet aggregation was induced by (**A**) ADP (5 µM final concentration), (**B**) thrombin receptor PAR-1-activating peptide TRAP-6 (20 µM), (**C**) epinephrine (2 µM), and (**D**) the TxA_2_ mimetic U46619 (3 µM) and monitored as % light transmission for 5 min.

To explain the index patient’s and his daughter’s bleeding diathesis, a comprehensive platelet function analysis was performed in comparison to his unaffected wife. Light transmission aggregometry demonstrated for both patients a defective secondary wave induced by ADP (Figure 2A), the thrombin receptor PAR-1 activating peptide TRAP-6 (Figure 2B), epinephrine (Figure 2C), and the TxA_2_ mimetic U46619 (Figure 2D), which was associated with partial disaggregation. This platelet aggregation defect was more pronounced for the index patient (hemizygous carrier of the *GATA1* variant) compared to his affected daughter (heterozygous carrier of the *GATA1* variant).

Normal platelet aggregation responses to all agonists were observed for the index patient’s unaffected wife, as expected, who served as a day control for all platelet function tests. Flow cytometric analysis of the main platelet surface receptors, i.e., αIIbβ3 integrin (CD41/CD61), GPIbα (CD42b), GPIX (CD42a), GPVI, and β1 integrin, showed normal expression profiles for the hemizygous and heterozygous carriers compared to the day control and additional healthy volunteers (Figure S3), ruling out the fact that the GATA1 T296P variant causes quantitative defects of these receptors.

### 3.6. The Index Patient and His Daughter Presented with a Platelet δ-Granule ATP Secretion Defect When Platelet Aggregation Was Nearly Normally Induced by High Concentrations of Collagen and Arachidonic Acid

**Figure 3:**
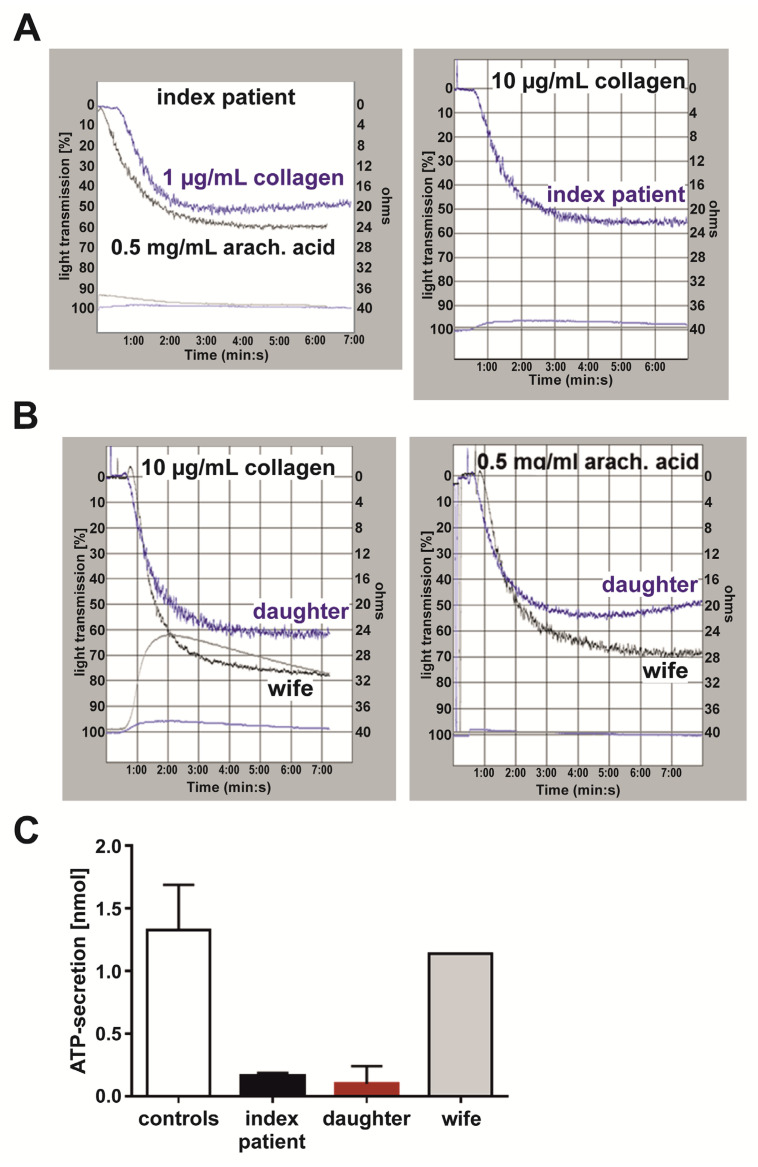
Simultaneous platelet aggregation and δ-granule ATP secretion capacity (index patient, daughter, and wife) induced by collagen and arachidonic acid. Platelets in platelet-rich plasma were adjusted to 2 × 10^8^/mL with autologous platelet-poor plasma, and simultaneous platelet aggregation and ATP secretion induced by collagen and arachidonic acid were monitored for 8 min by lumi-aggregometry. (**A**) Representative platelet aggregation (top curves, right *y*-axis light transmission (%)) and ATP release (bottom curves represent bioluminescence signals) of (**A**) index patient induced by 1 µg/mL, 10 µg/mL, and 0.5 mg/mL arachidonic acid, and (**B**) index patient’s daughter and wife (day control) induced by 10 µg/mL collagen and 0.5 mg/mL arachidonic acid, respectively. (**C**) Quantitation of platelet-secreted ATP in nmol for the index patient (black; *n* = 3, collagen (1 µg/mL, 10 µg/mL), arachidonic acid (0.5 mg/mL)), his daughter (red; *n* = 2, 10 µg/mL collagen, 0.5 mg/mL arachidonic acid), his wife (grey; *n* = 1, 10 µg/mL collagen), and healthy adult controls (white, *n* = 30, 10 µg/mL collagen, 0.5 mg/mL arachidonic acid). Data with *n* ≥ 2 are presented as mean ± SD.

Feedback activation processes through released ADP and ATP from exocytosed δ-granules importantly contribute to irreversible platelet aggregation, which is characterized by the second aggregatory wave [23]. To elucidate whether a potential δ-granule secretion defect could be the cause of the patients’ missing secondary aggregation response, lumi-aggregometry was performed for simultaneous monitoring of platelet aggregation and ATP secretion. Nearly normal aggregation responses of the index patient’s (Figure 3A) and his daughter’s (Figure 3B) platelets were observed for the strong platelet agonists collagen and arachidonic acid at high concentrations (collagen: 10 µg/mL, arachidonic acid: 0.5 mg/mL).

However, collagen- or arachidonic-acid-induced ATP secretion from platelet δ-granules was strongly reduced for both patients, as demonstrated by the flat bottom curves compared to the curve of the unaffected index patient’s wife (Figure 3B, left graph). Less than 0.25 nmol released ATP per 2 × 10^8^ platelets was quantified for both patients compared to controls with a lower limit of 0.98 nmol released ATP/2 × 10^8^ platelets (Figure 3C). These data indicate a severe δ-granule ATP secretion defect for the hemizygous and affected heterozygous carriers of the GATA1 T296P variant.

### 3.7. GATA1 T296P Variant Was Associated with a Combined Platelet α-/δ-Storage Pool Deficiency, Characterized with Reduced Numbers of α- and δ-Granules, Where Residual δ-Granules Lack ADP/ATP but Not Serotonin

**Figure 4:**
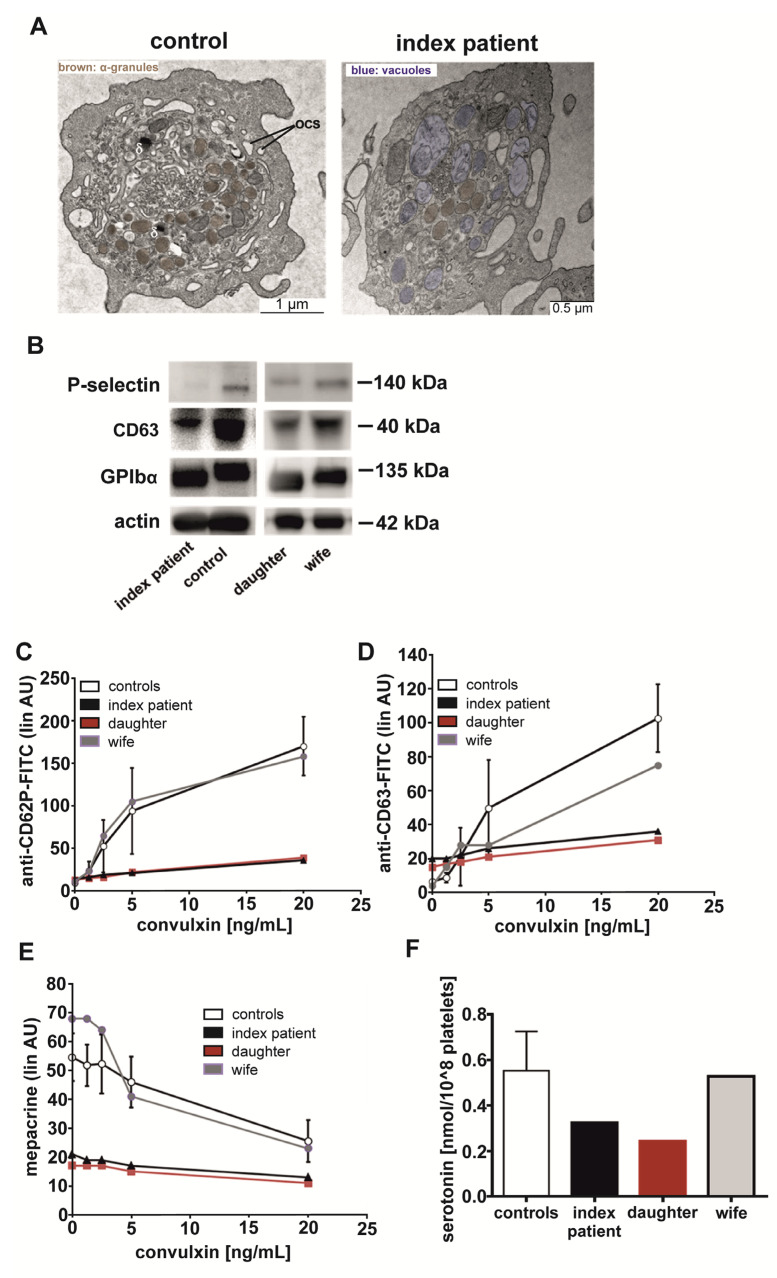
Platelet α- and δ-granule analysis of the GATA1-T296P-variant-affected index patient, his daughter, and his unaffected wife. (**A**) Transmission electron images of platelets from the index patient and his wife as day control. α-granules are colored in brown, δ-granules are indicated as δ; OCS: surface-connected or open canalicular system, where vacuole compartments are colored in blue. (**B**) Immunoblotting analysis of P-selectin (CD62P), CD63, and GPIbα in lysates of washed platelets from the index patient, his daughter, his wife, and an additional day control. Actin labeling served as protein loading control. (**C**,**D**) Flow cytometric analysis of (**C**) P-selectin/CD62P and (**D**) CD63 platelet surface expression in response to the GPVI-agonist convulxin of the index patient, his daughter, his wife, and additional controls (*n* = 30). (**E**) Flow cytometric analysis of mepacrine uptake in platelet δ-granules (convulxin 0 µg/mL) and convulxin-induced release convulxin of the index patient, his daughter, his wife, and additional controls (*n* = 30). (**F**) Quantification of serotonin in lysates of washed platelets from the index patient, his daughter, his wife, and additional controls (*n* = 10) by using an ELISA assay.

Platelet δ-granule ATP-secretion defects, as observed in both affected patients, are commonly caused by a δ-granule storage pool deficiency, characterized by quantitative and/or qualitative granule defects [23]. Electron microscopy analysis of thin sections of the index patient’s platelets showed a reduced number of δ-granules (counted 8 in 50 patient’s platelets vs. 85 δ-granules in 50 control platelets) but also of α-granules (counted 228 in 50 patient’s platelets with complete absence of a-granules in 2 of 50 patient’s platelets vs. 901 in 50 control platelets) compared to platelets from his wife as day control (α-granules colored in brown, δ-granules: black dots; Figure 4A). Furthermore, index patient’s platelets contained large vacuoles with partially included membrane/granule fragments (blue color, Figure 4A).

The reduced number of platelet α-granules was associated with a reduced immunoblot staining of the α-granule membrane marker P-selectin for the index patient compared to a day control as well as for the affected daughter compared to the index patient’s unaffected wife (Figure 4B). Accordingly, the reduction in the number of δ-granules was associated with reduced immunoblot staining of CD63 for both affected patients, which is predominantly expressed in the membrane of δ-granules and lysosomes. GPIbα, the major receptor subunit of the von Willebrand factor receptor complex GPIb-V-IX, was normally expressed in patients’ platelet lysates, as expected. These results are in line with strongly impaired platelet surface exposure of P-selectin (Figure 4C, CD62P) and CD63 (Figure 4D) for both affected patients induced by the collagen receptor GPVI agonist convulxin as assessed by flow cytometry. To check whether the residual δ-granules still contain their cargo, i.e., ADP/ATP and serotonin, the flow cytometric mepacrine assay and a serotonin ELISA were performed. In resting platelets, the cell membrane permeable green fluorescent dye mepacrine specifically binds to the δ-granule cargo molecules ADP and ATP, thereby inducing a significant fluorescence signal as detected for resting control platelets (Figure 4E, convulxin 0 µg/mL controls, wife). Convulxin mediated a dose-dependent extracellular release of ADP/ATP via δ-granule exocytosis of control platelets, leading to a dose-dependent decrease in the mepacrine fluorescence signal (Figure 4E, controls, wife). In contrast, the mepacrine signals in affected patients’ platelets were even lower ex vivo (convulxin 0 µg/mL) than fully activated control platelets and did not further decrease in response to convulxin (Figure 4E), indicating that residual δ-granules in patients’ platelets lack ADP/ATP. Conversely, the serotonin content in patients’ isolated platelets was decreased compared to controls but still present (Figure 4F). On the basis of these data, it is very likely that the hemizygous and affected heterozygous carrier of the GATA1 T296P variant suffered from a quantitative α- and δ-storage pool deficiency accompanied by ADP/ATP deficiency of residual δ-granules.

### 3.8. mRNA Profiling of GATA1-Regulated Genes in Whole Blood from the Index, His Wife, and His Daughter

**Figure 5:**
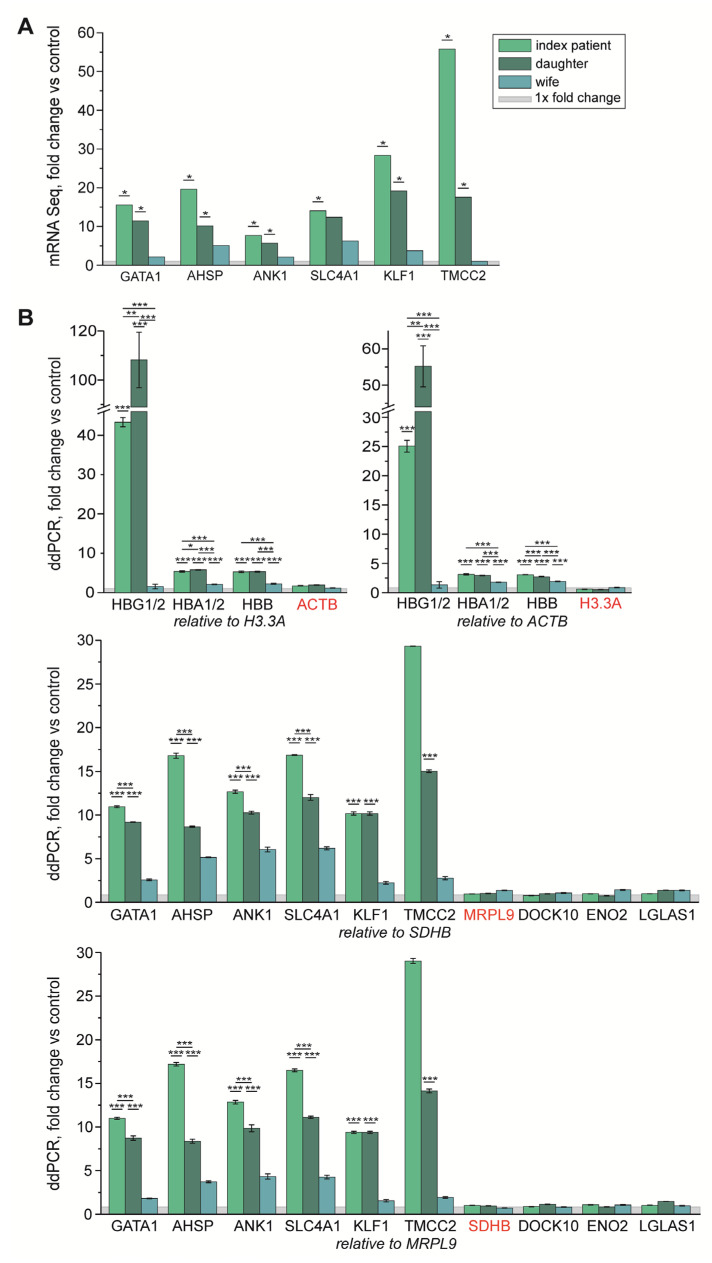
(**A**) Fold change vs. one control and of selected significantly deregulated genes detected in transcript profiling of the index patient (light green), his daughter (dark green), and his wife (blue). * = q < 0.05 (fitted for multiple testing). The 0–1-fold change is highlighted as a gray area. (**B**) ddPCR confirmation of significant expression differences noticed in NGS-based transcript profiling and of further potentially interesting genes missed by the analyses. Each measurement was set into relation to a housekeeper (*H3.3A, ACTB, MRPL9, SDHB*), and an additionally evaluated control gene is highlighted in red. The fold change was chosen for easy-to-understand representation, but the statistical significance levels shown were calculated on the basis of the relative transcription levels of the respective individuals set in comparison to three control subjects. Statistically significant differences in control genes were defined as naturally occurring and used as a detection limit. * = *p* < 0.05, ** = *p* < 0.01, *** = *p* < 0.001, light green = index patient, daughter = dark green, wife = blue, gray = 1× fold change.

To obtain further evidence for the pathophysiological significance of GATA1 T296P, we studied the mRNA expression of known GATA1-regulated genes. For this purpose, the transcriptome from whole blood of the index, his wife, and his daughter was analyzed (NGS-based RNA profiling) and compared to one healthy control, revealing 61 significantly (*p* < 5 × 10^−5^; level of significance after correction for multiple testing: q < 0.05) regulated genes in the index, 57 in the daughter, and none in the index’s wife (Table S6). A total of 35 genes were significantly regulated in the same manner in both patients. To identify the most interesting genes, the significantly regulated genes were filtered by two lists: (i) a list of exemplary GATA1 targets and important proteins of the red cell lineage (48 genes) and (ii) a list from a proteomics approach (data not shown) consisting of genes with strongly reduced protein expression in the platelets of the index patient (46 genes). Filtering of the mRNA profiling results revealed upregulation of *GATA1* and four further genes from list (i) in both patients, which have been consistently reported as typical GATA1 targets in erythrocytes: *AHSP*, *ANK1*, *KLF1*, and *TMCC2* (Figure 5A). Increased *GATA1* expression in the presence of a partial defect (absence of BCAM protein) indicates a compensatory response to the defect caused by T296P. The only difference between the index and his daughter was the *SLC4A1* transcript, which was only significantly upregulated in her (Figure 5A). Due to excessive expression and problems aligning the sequenced fragments to the reference transcripts, most hemoglobin genes (among them, *HBB, HBA1, HBA2, HBG1*, and *HBG2*), although interesting, had to be excluded from the evaluation.

The genes of list (ii) (proteome analysis) showed no changes in whole blood except for *TMCC2*. *TMCC2* is the only gene present in list (i) and (ii), and was not decreased in erythrocytes, as seen in platelets of the index, but markedly increased. This is plausible since the transcription factor network that defines the way and the extent of gene regulation by GATA1 differs markedly in thrombopoiesis and erythropoiesis.

ddPCR was used to confirm the results of the mRNA profiling and to reevaluate the non-analyzable hemoglobin genes. All mRNA profiling results could be confirmed (Figure 5B). Since the ddPCR has a higher sensitivity, significantly increased expression of *SLC4A1* was also detected in the index case. The wife, who did not have any changes in her mRNA profile, showed marked changes for *SLC4A1*, *ANK1*, and *AHSP* in ddPCR, although to a much lesser extent than the index and daughter, suggesting a mild erythrocyte defect and substantiating the pathogenicity of *SLC4A1* A737V. Exemplary genes from list (ii) were confirmed to be expressed normally. The sum signals for *HBB* and *HBA* transcripts were significantly upregulated in all three family members. The most pronounced and significant upregulation could be seen in *HBG* transcripts (particularly evident in the daughter), which matched the HbF quantification of the family by hemoglobin HPLC.

## 4. Discussion

This is the first report of a family with a X-linked GATA1-related bleeding disorder caused by a novel missense variant, c.886A>C, leading to the amino acid substitution T296P, which is located C-terminally to the second zinc finger domain of the megakaryopoiesis- and erythropoiesis-relevant transcription factor GATA1. The hemizygous male index patient and his heterozygous daughter with non-random X-chromosome inactivation were characterized with large platelets and a reduced number of α- and δ-granules, where the residual δ-granules lack ADP/ATP but not serotonin. However, the index patient but not his affected daughter suffered from moderate thrombocytopenia. Both patients presented with mild dyserythropoiesis, but a further maternally transmitted heterozygous *SLC4A1* c.2210C>T p.A737V variant in the daughter was identified as a potential disease modifier for mild spherocytosis and hemolysis.

**Figure 6:**
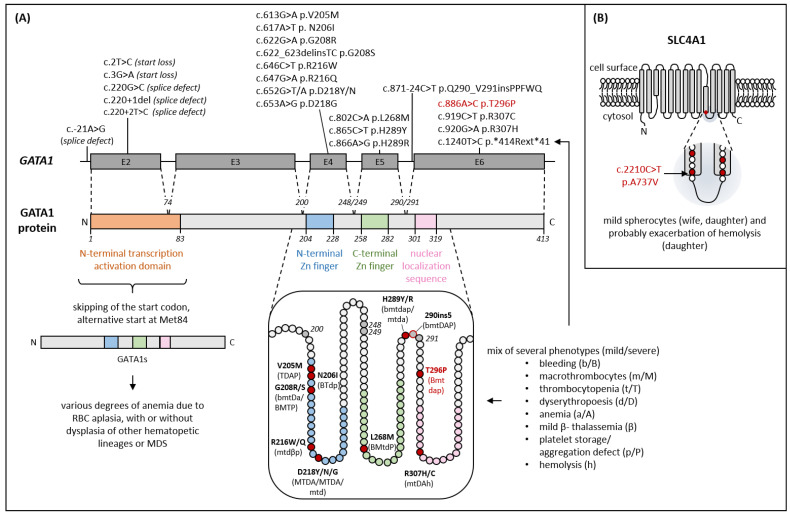
(**A**) Schematic representation of germline *GATA1* variants (NM_002049.4) with available functional analyses. The newly identified variant c.886A>C p.T296P is highlighted in red. Variants upstream and in exon 2 led to a skip of the actual start codon and used Met84 as a new start codon, resulting in a truncated GATA1, the GATA1s. All other variants cause different phenotypes, which are described by the letters in brackets in the enlarged representation of the region. [28,29,42,45,46,47,48,49] The letters correspond to the phenotypes listed next to it and the severity is symbolized by upper or lower case (lower case = mild, upper case = severe). In the enlarged view, the amino acids are shown as circles (red fill or border = mutated; gray fill = exon border). The illustration is merely schematic and does not represent secondary structures. (**B**) Transmembrane helices of SLC4A1 (NM_000342.4). The newly discovered variant A737V is located at the end of the 10th transmembrane helix of SLC4A1, and other variants known to cause hereditary spherocytosis or stomatocytosis in the close vicinity are highlighted (red circles) [36].

A number of distinct pathogenic *GATA1* variants have been identified within the first N-terminal zinc finger domain of GATA1 associated with variable macrothrombocytopenia, platelet aggregation defects, and α-granule deficiency (Figure 6A) [8,9,11]. However, there are only very few studies reporting a reduced number of both platelet α-granules and δ-granules in GATA1-related thrombocytopenia. Ultrastructural analysis of platelets from a patient with the GATA1 R216Q variant, causing macrothrombocytopenia with β-thalassemia, demonstrated a severe reduction of δ-granules and a paucity of α-granules, whereas macrothrombocytes presented with giant α-granules [37]. Germline defects affecting the length of GATA1 at the N- or C-terminus are described to be associated with α-/δ-storage pool deficiency. The synthesis of only the short GATA1s isoform caused by the splice variant c.220G>C (traditional name: 322G>C [38]) in exon 2 resulted in X-linked anemia with neutropenia, and two of seven affected males presented with a reduced number of α- and δ-granules and impaired ADP-, epinephrin-, and collagen-induced platelet aggregation [39]. A *GATA1* missense *414Rext*41 variant in the C-terminal termination codon leading to a X-linked blood group Lu(a-b-) phenotype with mild bleeding and mild macrothrombocytopenia was reported to be associated with paucity of α- and δ-granules and absent second wave of ADP- and epinephrine-induced platelet aggregation in one affected male [40]. A 5 aa insertion at the C-terminus of the C-terminal zinc finger of GATA1 as a result of an intronic splice variant affected two unrelated males with dyserythropoietic anemia, whereas for one patient with clinical bleeding, defective platelet aggregation in response to collagen, ADP, epinephrine, and arachidonic acid and severely impaired surface expression of the α-granule membrane marker P-selectin and the δ-granule/lysosome membrane marker CD63 in response to thrombin were shown [41]. A recently described L268M variant localized in the C-terminal zinc finger of GATA1 was associated with δ-granule paucity, mild alpha-granule defect, and increased levels of MYH10 in platelets from two child brothers [42].

In line with these reports, we observed for both affected patients impaired platelet aggregation with an absent second wave in response to ADP, epinephrine, TRAP-,6 and TxA_2_ mimetic; absent collagen and arachidonic acid-induced ATP-release; and strongly reduced P-selectin and CD63 surface expression in response to the collagen receptor GPVI agonist convulxin. Electron microscopy demonstrated a reduced number of platelet α- and δ-granules in the index patient and immunoblot analysis supported reduced levels of the α-granule membrane protein P-selectin and delta-granule/lysosome membrane protein CD63, indicating a combined quantitative α-/δ-storage pool deficiency. Interestingly, the residual δ-granules lacked ADP/ATP as assessed by the flow cytometric mepacrine uptake assay and lumi-aggregometry but still contained serotonin, supporting an additional differential qualitative defect of the δ-granules affecting adenine nucleotides. Thus, this so-called AN-subtype of δ-storage pool deficiency [43] was observed for the first time in these affected patients with GATA1-related disorders. However, whole mount electron microscopy, the appropriate method and gold standard for the quantification of δ-granules, was not applied, which represents a limitation of this study. Furthermore, analysis of the a-granule membrane protein P-selectin is rather an indirect marker for quantitative a-granule defects because shedding of P-selectin from the platelet surface can occur upon platelet activation [44]. To prevent artificial platelet activation ex vivo, blood collection was performed simultaneously from patients and day controls in a standardized manner and processed within one hour [17].

GATA1 is involved in controlling the expression of megakaryopoiesis-relevant genes, e.g., *GPIBA*, *GPIBB*, *GP9*, and *NBEAL2*, whose encoding proteins are crucial for platelet function as well. Freson and coworkers demonstrated in affected family members with the GATA1 D218G/Y variants within the N-terminal zinc finger loop defective transcription of the *GPIBA*, *GPIBB*, and *GP9* genes, leading to a fraction of enlarged platelets lacking the von Willebrand factor receptor complex GPIb-V-IX, including GPIbα and GPIbβ, and impaired ristocetin-induced platelet agglutination [50,51]. In a male patient with the GATA1 N-terminal zinc finger variant R216Q, a decrease in expression of the collagen receptor GPVI was detected, associated with impaired platelet aggregation and tyrosine phosphorylation in response to submaximal collagen concentration [52]. In our affected patients with a GATA1-defect closely located to the C-terminal zinc finger, no alterations of the major platelet surface receptors, i.e., αIIbβ3 integrin, GPIbα, GPIX, β1 integrin, GPVI, and CD36, were observed by flow cytometric analysis. Normal expression of these receptors was supported by LC–MS/MS-based proteome analysis of the index patient’s platelets compared to four unrelated controls (data not shown). These data substantiate our conclusion that rather granule defects than alteration in receptor expression cause the observed platelet dysfunction in our patients. Freson and coworkers also reported for two patients with macrothrombocytopenia that the GATA1 N-terminal zinc finger variants D218Y/G suppress *NBEAL2* RNA and protein expression, leading to paucity of platelet α-granules. However, proteomic mass spectrometry analysis did not reveal lower NBEAL2 expression in our index patient compared to controls (data not shown).

Distinct pathogenic variants in the N-terminal zinc finger (aa204–228) have been shown to cause maturation defects not only of megakaryocytic but also erythroid precursors [47,48]. Careful investigation of the mutation specific impairment of different lineages in patients with pathogenic germline (Figure 6A) or somatic *GATA1* variants has contributed crucially to elevate the understanding of complex GATA1-regulated mechanisms [47]. Those pathogenic variants therefore mediate diverse clinical consequences. Symptoms related to erythropoiesis range from clinically unremarkable to mild anemia with dyserythropoiesis or severe transfusion-dependent anemias. Their red cell indices are usually characterized by normal MCH and normal to elevated MCV (100–103 fl) [48]. For one variant, however, β-thalassemia-like symptoms, including microcytosis and hemoglobin chain imbalance with elevated HbA2 and HbF levels, have been described [53].

In the C-terminal GATA1 zinc finger (aa258–282, Figure 6A), which mainly mediates the DNA-binding activity of GATA1, only one pathogenic variant has been detected so far (L268M), causing a bleeding phenotype due to a severe platelet defect but no anemia [42]. In the nearby nuclear localization sequence (aa301–319), two pathogenic base exchanges affecting the same amino acid have been reported (R307H/C), but with the main symptom being congenital hemolytic anemia [14] or severe fetal anemia requiring intrauterine transfusion [46]. The region between the C-terminal GATA1 zinc finger and the nuclear localization sequence is vastly uncharacterized, as only two variants, affecting two adjacent amino acids, have been observed (H289Y/R and Q290-V291ins5) [28,29]. Therefore, the here-reported family with an index hemizygous for T296P offers the opportunity to further characterize the role of this region in erythropoiesis. The variant caused prominent platelet-related bleeding symptoms, while anemia was only borderline and clinically silent. Routine laboratory testing revealed mild macrocytic and hyperchromic red blood cells, reticulocytosis, elevated HbF levels, and elevated ferritin without acute phase (normal CRP) with otherwise largely normal erythrocyte morphology and normal HbA2, all suggestive of mild dyserythropoiesis. These findings match those of the patients with H289Y/R, which show either a bleeding disorder due to impaired platelet aggregation and a mild macrocytic anemia or only mild macrocytic anemia [28,29], but differ from a patient with Q290-V291ins5 who has marked dyserythropoietic anemia. The similar clinical consequences of T296P and H289Y/R suggest a region-specific symptom pattern, possibly modulated by the exact amino acid exchange.

The rare Lu(a-b-) blood group phenotype of our index case is caused by hampered expression of the GATA1-dependent *BCAM* gene. Even though Lu(a-b-) has already been reported in a patient who is hemizygous for a stop loss variant (p.*414Rext*41, [40]), Lu blood group antigens were not studied in most other patients with GATA1 defects. Therefore, assignment of Lu negativity to a specific region or specific variants in GATA1 is difficult. However, the absence of Lu antigens may present an easy-to-analyze marker for the confirmation of a GATA1 defect (at least affecting *BCAM*) in a routine setting.

mRNA profiling revealed a set of deregulated genes known to be GATA1 dependent (*AHSP, ANK1, SLC4A1, KLF1, TMCC2, HBG1/2, HBB, HBA1/2*) and confirmed a relevant GATA1 defect in erythrocytes. Most strikingly, *HBG1/2* transcripts were massively overexpressed in the index. Although *HBB* transcripts were upregulated, the extent of elevation was much greater for *HBG1/2* transcripts, indicating an incomplete switch from γ-globin to β-globin chains, which is characteristic for a GATA1 defect. The artificial impairment of this switch is currently being discussed as a potential therapeutic strategy in hemoglobin β-chain diseases [54].

Heterozygous female *GATA1* variant carriers are usually asymptomatic, suggesting a recessive inheritance of *GATA1* mutations [10,48]. However, close examination of platelets revealed single macrothrombocytes in the female carriers [55]. X-inactivation studies in families with substitutions of amino acid 218 showed either random X-inactivation or a shift in favor of the reference allele, suppressing the variant [51]. Apart from the daughter described in this study, only one other observation of a symptomatic female could be found, but unfortunately investigation of X-inactivation was missing [56]. The daughter of our family presented with significant bleeding symptoms. She did not show thrombocytopenia but macrothrombocytes and the same but less severe functional platelet defect as her father. However, she had more evidently elevated HbF levels; borderline, impaired BCAM expression (Lu(a-b+) with low strength Lu^b^); and similarly deregulated GATA1-dependent genes as her father. In contrast to the published female carrier with D218N, her X-inactivation was skewed towards the mutant allele (only ≈10% reference allele activity), which is why a significant GATA1 activity impairment was also seen in the heterozygous daughter. In contrast, her red blood cell indices in the lower reference range and the detection of spherocytes and hemolysis differentiate her phenotype form that of the father.

The analysis of the asymptomatic mother’s blood count, however, revealed similar abnormalities. She had borderline anemia, microcytosis, subtly elevated HbF, and spherocytes in the blood smear, but hemolysis was not observed. Detection of the *SLC4A1* c.2210C>T p.A737V variant (Figure 6B) suggested mild, clinically largely silent spherocytosis in both the mother and daughter. While the expression of *GATA1* and *KLF1* were not significantly increased in the mother, markedly increased expression levels were found for *SLC4A1* and *ANK1*, which is consistent with compensatory upregulation of these genes due to a mild SLC4A1 defect. Thus, we hypothesize that the coexistence of the *GATA1* and *SLC4A1* variant causes a more evident impairment of erythropoiesis (hemolysis, high HbF) in the daughter compared to her father. Therefore, the *SLC4A1* variant can be regarded as a modifier of the paternally transmitted GATA1 defect in the daughter. Similar combinations of GATA1 variants and exchanges affecting other erythrocytic genes that aggravate the GATA1-caused phenotype have been described. For example, a boy hemizygous for GATA1 H289R had a significantly worse outcome in contrast to other affected male relatives due to a heterozygous pyruvate kinase variant [28]. The co-inheritance of a GATA1 G208R with an ALAS2 R479Q led to a shift of the *GATA1*-typical anemia towards a sideroblastic anemia with macrothrombocytopenia [57].

In conclusion, our study presents new insights in how *GATA1* missense variants C-terminally located to the second zinc finger domain can affect platelet phenotype and function and erythropoiesis in hemizygous and heterozygous carriers and that a heterozygous *SLC4A1* c.2210C>T p.A737V variant compounds mild dyserythropoiesis to mild spherocytosis and hemolysis.

## Figures and Tables

**Table 1:** Laboratory characteristics of the index patient (male, age 36), his daughter (female, age 4), and his wife (female, age 30).

Analyte		Index	Daughter	Wife	Reference	Unit
White blood cell count		7.5	6.80 (5.5–15.5)	6.20	3.5–10	×10^3^/µL
Automated differential blood count		normal	normal	normal	normal	-
Red blood cell count		**−3.70** (4.3–6.3)	3.63 (3.1–5.2)	4.07 (3.7–4.8)	indiv.	×10^6^/µL
Platelets		**−88**	178.0	273.0	150–360	×10^3^/µL
Mean platelet volume		10.4	11.2	9.9	7.6–11.2	fL
Hemoglobin		**−12.5** (13.5–17.5)	10.9 (10–13)	**−11.6** (12–16)	indiv.	g/dL
Hemoglobin HPLC	HbF	**+2.8**	**++13.5**	**+1.8**	<1	%
	HbA2	3.0	2.2	2.7	<3.3 (β-thalassemia)	%
	Variant hemoglobin	none	none	none	none	-
Hematocrit		**−37.2** (39–49)	31.8 (30–45)	**(−)33.5** (34–44)	indiv.	%
Mean corpuscular volume		**(+)100.3**	87.6	**(−)82.3**	83–100	fL
Mean corpuscular hemoglobin		**(+)33.7**	30.0	28.5	27–33	pg
MCHC		33.6	34.3	34.6	32–35	g/dL
RDW-CV		14.3	13.9	14.2	11–15	%
Blood smear	White blood cells	normal	normal	normal	normal	-
	Red blood cells	normal	**+ spherocytes**	**++ spherocytes**	normal	-
	Platelets	**macrothrombocytes**	**macrothrombocytes**	normal	normal	-
C-reactive protein		2.2	0.20	n.d.	<5	mg/L
Ferritin		**+332** (20–275)	**+35** (6–24)	9.4 (5–200)	indiv.	ng/mL
Transferrin saturation		**−15.1**	38.9 (7–44)	**−−7.4**	16–45	%
Haptoglobin		0.84 (0.14–2.58)	**−<0.08** (0.11–2.2)	1.69 (0.35–2.50)	indiv.	g/L
Zinc protoporphyrin		75	80	**(+)104**	>100 (iron deficiency)	mmol/mol heme
Reticulocytes abs.		**+210**	**+114**	**+78**	19–69	/nL
Erythropoietin		19.1	**+37.1**	13.2	4.3–29	mU/mL
Lutheran blood group		**a-b-**	a-b+	a-b+	a-b+	-

(xyz) = individual reference values; indiv. = individual reference values; MCHC = mean corpuscular hemoglobin concentration; RDW_CV = variation coefficient of red cell distribution width; bold = values outside the reference range; “-“, “—“, “+” or “++” = values moderately (single symbol) or far (double symbol) outside of the reference range; (+) or (−) = values at the upper or lower reference limit.

**Table 2:** Comparison and classification of filtered potentially disease-causing variants detected by exome sequencing.

		*GATA1*	*EPHB1*	*AP3B1*	*SLC4A1*
		c.886A>C	c.1856T>C	c.1069A>G	c.2210C>T
		p.(Thr296Pro)	p.(Val619Ala)	p.(Ile357Val)	p.(Ala737Val)
Reference sequence		NM_002049.4	NM_004441.5	NM_003664.5	NM_000342.4
Chromosome		X	3	5	17
Exon		6	10	10	17
dbSNP		no entry	rs375222902	rs142025324	rs886052997
ClinVar (number of entries)		no entry	no entry	VUS (2), likely benign (4)	VUS (3)
MAF in GnomAD		no entry	0.0001532	0.001795	0.000003982
Mutation Taster		disease causing	disease causing	disease causing	disease causing
PolyPhen2		probably damaging	probably damaging	benign	probably damaging
SIFT		deleterious	deleterious	tolerated	deleterious
PROVEAN		deleterious	deleterious	neutral	deleterious
OMIM Gene		305371	600600	603401	109270
OMIM Disease		300367 (XLTDA)	no entry	608233 (HPS2)	612653 (SPH4)
Inheritance (OMIM)		XLR	no entry	AR	AD
ACMG criteria		pathogenic	VUS	likely benign	pathogenic
Variant-related symptoms in the affected family members		macrothrombocytopenia, mild dyserythropoesis	none	none	mild spherocytosis, potential modification of GATA1 disease
NGS result confirmed		yes (pyrosequencing)	yes (Sanger sequencing)	yes (Sanger sequencing)	yes (Sanger sequencing)
Part of in-house coagulation panel (322 genes)		✓	✓	✓	-
Part of in-house anemia panel (96 genes)		✓	-	-	✓
Genotype	index (bleeding)	C	TC	AG	CC
	wife (spherocytes)	AA	TT	AA	CT
	daughter (bleeding, spherocytes, hemolysis)	AC	TC	AG	CT
	son 1 (healthy)	A	TT	AA	CC

VUS = variant of unknown significance; MAF = minor allele frequency; XLTDA = X-linked thrombocytopenia with/without dyserythropoietic anemia; HPS2 = Hermansky–Pudlak syndrome 2; SPH4 = hereditary spherocytosis type 4; XLR = X-linked recessive inheritance; AR = autosomal recessive inheritance; AD = autosomal dominant inheritance; ✓ = gene is part of this inhouse panel.

## Data Availability

All relevant data generated or analyzed during this study are included in this published article or its Supplementary Materials. Restrictions apply to the availability of the complete next-generation sequencing data of the patients to preserve patient confidentiality. The corresponding author will on request detail the restrictions and any conditions under which access to some data may be provided.

## Supplementary Materials

The following supporting information can be downloaded at https://www.mdpi.com/article/10.3390/cells11193071/s1, Supplementary methods: Table S1: Primers used to confirm whole exome sequencing results and screen additional family members via Sanger sequencing; Table S2: Primers used to confirm whole exome sequencing results and screen additional family members via pyrosequencing; Table S3: Primers used for pyrosequencing of X-chromosomal polymorphisms and GATA1 pathogenic variant; Table S4: Primers used for ddPCR analyses of whole blood mRNA. And Supplementary results: Figure S1: Pyrograms of *GATA1* c.886A>C p.(Thr296Pro) (exon 6, NM_002049.4); Figure S2: Electropherograms of *SLC4A1* c.2210C>T p.(Ala737Val) (exon 17, NM_000342.4) of the index case, his wife and daughter (blue vertical line = base exchange); Figure S3: Flow cytometric analysis of major platelet surface receptors from GATA1 index patient, affected daughter, unaffected wife and additional healthy controls; Table S5: Blood count of the father, mother and son 1. The reference values are given in brackets; Table S6: Transcripts significantly differentially expressed in index case or daughter (overlapping transcripts highlighted in grey).
