# Supplementary material for: Novel GATA1 Variant Causing a Bleeding Phenotype Associated with Combined Platelet α-/δ-Storage Pool Deficiency and Mild Dyserythropoiesis Modified by a SLC4A1 Variant
Source: Cells. 2022 Sep 29;11(19):3071. doi: 10.3390/cells11193071 (PMC9564339; doi:10.3390/cells11193071)
Supplement: Supplementary file 1 [file cells-11-03071-s001.zip › cells-1925428-supplementary.pdf]

cells

MDPI

Supplementary Material

Supplemental Methods **2**

Exome Sequencing 2

X-Inactivation Analysis 3

mRNA Sequencing 4

ddPCR 4

Supplemental Results **5**

Blood count of additional family members 5

Pyrosequencing of GATA1 c.886A>C p.(Thr296Pro) 6

Sanger sequencing SLC4A1 c.2210C>T p.(Ala737Val) 7

Surface presentation of major platelet receptors 8

mRNA profiling results 9

Supplementary Methods

Exome Sequencing

Coagulation disorder panel (322 genes):

- ABCA1, A2M, ABCG5, ABCG8, ACVRL1, ADAMTS13, ADCY3, ADCY6, ADCY7, ADORA2B, ADRA2A, AKT1, ALOX12, ANKRD26, ANO6, ANXA2, ANXA3, ANXA5, AP3B1, AP3D1, AP3M1, AP3S1, ARHGAP1, ARHGAP17, ARHGAP32, ARHGAP6, ARHGDIA, ARHGDIB, ARHGEF12, ARRB1, B4GALNT2, BLOC1S1, BLOC1S2, BLOC1S3, BLOC1S4, BLOC1S5, BLOC1S6, BTK, C1QA, C4BPA, C4BPB, CD177, CD36, CFH, CFI, CHST14, CLEC1B, COL1A1, CPB2, CSK, CTTN, CYCS, DAAM1, DIAPH1, DIAPH2, DNM2, DTNBP1, EFNB1, ENG, ENTPD1, EPHA4, EPHB1, F10, F11, F12, F13A1, F13B, F2, F2R, F2RL3, F3, F5, F7, F8, F9, FARP2, FCGR2A, FERMT1, FERMT3, FGA, FGB, FGD3, FGG, FGR, FHOD1, FLI1, FLNA, FN1, FYN, GATA1, GDI2, GFI1B, GGCX, GNA12, GNA13, GNAI1, GNAI2, GNAQ, GNAZ, GNB2, GNB3, GNG11, GNG12, GNG13, GNG5, GP1BA, GP1BB, GP5, GP6, GP9, GRAP2, GRB2, GRK2, GRK5, GRK6, GUCY1A3, GUCY1B3, HOXA11, HPS1, HPS3, HPS4, HPS5, HPS6, HRG, HTR2A, INPP5D, ITGA2, ITGA2B, ITGA5, ITGB1, ITGB3, ITPR1, JAK2, KLKB1, KNG1, LAIR1, LAT, LCP2, LEFTY2, LMAN1, LTBP1, LY6G6F, LYN, LYST, MAPK1, MAPK13, MAPK14, MAPK8, MASTL, MCFD2, MLPH, MMRN1, MMRN2, MPL, MTHFR, MYH9, MYLK, MYLK2, MYO5A, NAPA, NAPG, NBEA, NBEAL2, NIPSNAP3A, NSF, ORAI1, P2RX1, P2RY1, P2RY12, PAFAH1B1, PDE2A, PDE3A, PDE4D, PDE5A, PDGFA, PDGFB, PDGFC, PDGFD, PDGFRA, PDGFRB, PDPK1, PEAR1, PF4, PIK3CA, PIK3CB, PIK3CD, PIK3CG, PIK3R1, PIK3R3, PIK3R5, PLA2G2A, PLA2G4A, PLA2G4C, PLA2G7, PLAT, PLAU, PLAUR, PLCB2, PLCB3, PLCG2, PLG, PPP1CA, PPP1CB, PPP1CC, PPP1R12A, PPP1R12C, PPP1R14A, PPP1R2, PRKACA, PRKACB, PRKACG, PRKAR1A, PRKAR2A, PRKCA, PRKCB, PRKCD, PRKCQ, PRKD1, PRKG1, PRKG2, PROC, PROCR, PROS1, PROZ, PTEN, PTGIR, PTGS1, PTGS2, PTK2, PTPN11, PTPN6, PTPRA, PTPRJ, RAB27A, RAB27B, RAB38, RAB4A, RABGGTA, RAC1, RAP1GAP, RAP1GAP2, RAP1GDS1, RASGRP2, RBM8A, RGS10, RGS18, RGS19, RGS20, RGS9, RHOA, RHOC, RHOF, ROCK1, ROCK2, RUNX1, SCAMP2, SCFD1, SELP, SEPT5, SERPINA1, SERPINA10, SERPINA2, SERPINA5, SERPINC1, SERPIND1, SERPINE1, SERPINE2, SLC35D3, SLC9A3R1, SMAD4, SNAP23, SNAP25, SNAP29, SNAPIN, SNX1, SRC, STIM1, STOM, STX11, STX12, STX2, STX4, STX6, STX7, STXBP3, SYK, SYTL4, TBXA2R, TBXAS1, TEC, TET2, TFPI, TFPI2, TGFB1, TGFB3, THBD, THPO, TLN1, TLR2, TREML1, TTC37, TUBB1, UNC13A, VAMP2, VAMP3, VAMP7, VAMP8, VAV1, VAV2, VAV3, VKORC1, VPS11, VPS16, VPS18, VPS33A, VPS33B, VPS39, VPS41, VWF, WAS, WIPF1, WIPF2, WIPF3

Anemia panel (96 genes):

- ABCG5, ABCG8, ADA, AK1, ALDOA, ANK1, BLVRB, BRCA2, BRIP1, CYB5A, CYB5R3, DKC1, EPB41, EPB42, ERCC4, FANCA, FANCB, FANCC, FANCD2, FANCE, FANCF, FANCG, FANCI, FANCL, FANCM, G6PD, GAPDH, GATA1, GCLC, GOT1, GPI, GSR, GSS, HAMP, HBA1, HBA2, HBB, HBD, HBE1, HBG1, HBG2, HBZ, HFE, HJV, HK1, NHP2, NOP10, NT5C3A, PALB2, PARN, PFKL, PFKM, PGD, PGK1, PIEZO1, PKLR, RAD51C, RPL11, RPL15, RPL26, RPL27, RPL31, RPL35A, RPL5, RPS10, RPS17, RPS19, RPS24, RPS26, RPS27, RPS28, RPS29, RPS7, RTEL1, SBDS, SLC11A2, SLC40A1, SLC4A1, SLCO1B1, SLCO1B3, SLX4, SPTA1, SPTB, STEAP3, TERC, TERT, TFR2, TINF2, TMPRSS6, TPI1, TSR2, UGT1A1, UGT1A6, , UGT1A7, UROS, WRAP53

Platelet proteomic analysis of index patient (46 genes):

- QSOX1, PPP6C, TMCC2, HSPA4L, FTH1, CSTB, PRNP, LPL, SERPINE2, ENO2, LGALS1, P4HA1, ST6GAL1, ITGB5, MAOB, CTGF, ALDH4A1, GRK6, GSTM5, CXCL12, SELENOP, SULT1A1, PPT1, SLC12A2, STXBP1, SELENOT, MAP1A, FHL2, SEC23B, FNBP1L, STEAP3, CD109, RINL, ERO1B, TDRP, C2CD5, DOCK10, RARRES2, P2RY12, FN3K, PLXNA4, DECR2, SPHK1, CEP131, WASF3, MRVI1

Table S1. Primers used to confirm whole exome sequencing results and screen additional family members via Sanger sequencing.

Gene	Exon	Forward Primer (5′-3′)	Reverse Primer (5′-3′)	Prod. Length [bp]	Tm [°C]
EPHB1	10	*GTAAAACGACGGCCA*GATGAGGAGGCCCAGCAAGAG	*CAGGAAACAGCTATGAC*AGGGTGGTGGAAAGAAGATGTT	348	63.0/60.0
AP3B1	10	*GTAAAACGACGGCCA*AGTGCTTACTCAAAGATGTTTCATTG	*CAGGAAACAGCTATGA*CGGTTCAAACATCCCCTGGATTA	217	57.3/60.9
SLC4A1	17	AGGAGGCAGGGGAGAACC	ATGTGGGGAAGTGGTGCA	429	60.5/60.0

Italic = M13 sequencing tag.

Table S2. Primers used to confirm whole exome sequencing results and screen additional family members via pyrosequencing.

Gene	Exon	Forward Primer (5′-3′)	Reverse Primer (5′-3′)	Sequencing Primer (5′-3′)	Prod. Length PCR [bp]	Tm [°C]
GATA1	6	Bio-TTGACACAGAGAGGCAAAGGT	TTCTTTTTCCCTTTTCCAGATGC	CATCCTTCCGCATGG	135	58.8/61.6

Bio = 5′ biotinylated primer.

X-Inactivation Analysis

Table S3. Primers used for pyrosequencing of X-chromosomal polymorphisms and GATA1 pathogenic variant.

<table>
<tr><th>Gene (type)</th><th>Exon</th><th>Forward Primer (5′-3′)</th><th>Reverse Primer (5′-3′)</th><th>Sequencing Primer (5′-3′)</th><th>Prod. Length PCR [bp]</th><th>Tm [°C]</th></tr>
<tr><td>GATA1 (gDNA)</td><td>6</td><td>Bio-TTGACACAGAGAGGCAAAGGT</td><td rowspan="2">TTCTTTTTCCCTTTTCCAGATGC</td><td rowspan="2">CATCCTTCCGCATGG</td><td>135</td><td>58.8/61.6</td></tr>
<tr><td>GATA1 (cDNA)</td><td>6</td><td>Bio-TGCGGCCTCTACTACAAGCT</td><td>104</td><td>59.1/61.6</td></tr>
<tr><td>ELF4 (gDNA)</td><td>12</td><td>TCAGTGGCCTCCCCCAAC</td><td rowspan="2">Bio-TGAAGGCAGCAATGACAGTCC</td><td rowspan="2">AACCCGGCGCCACCC</td><td>126</td><td>63.5/62.6</td></tr>
<tr><td>ELF4 (cDNA)</td><td>12</td><td>TACCTCCACCATGCTCGTCTCT</td><td>446</td><td>62.3/62.6</td></tr>
<tr><td>PRPS2 (gDNA)</td><td>1</td><td rowspan="2">CTGTTCAGCGGCAGCTCG</td><td>Bio-CTGGTCTCCTGGTTGCTGAACT</td><td rowspan="2">AGCTCGCATCAGGAC</td><td>107</td><td>63.5/61.9</td></tr>
<tr><td>PRPS2 (cDNA)</td><td>1</td><td>Bio-TCGGGCGTATGGGAAACAC</td><td>273</td><td>63.5/63.0</td></tr>
</table>

Bio = 5′ biotinylated primer.

mRNA Sequencing

List of GATA1-regulated genes, used to filter mRNA profiling data (49 genes):

- ACKR1, AHSP, AIFM2, ALAD, ALAS2, ANK1, AQP1, ARG1, ART4, BCAM, BSG, CD44, EKLF, ELANE, EPB42, EPOR, ERAP1, ERMAP, FCER1A, FOG1, G6PD, GATA1, GYPA, HBA1, HBA2, HBB, HBE1, HBG2, HLA-A, HLA-B, HLA-DPB1, HLA-DRA, HLA-DRB1, HLA-DRB4, HLA-G, IL4R, KLF1, MPO, NCF4, NFE2, PKLR, RNASE2, RNASE3, SLC4A1, TMOD1, TPSAB1, TPSAB1, TPSB2, UROD

ddPCR

Table S4. Primers used for ddPCR analyses of whole blood mRNA.

Gene	Exon	Forward Primer (5′-3′)	Reverse Primer (5′-3′)	Prod. Length [bp]	Tm [°C]
SLC4A1	7–8	CAACACTCCTCACTGGAGACAC	GAATTCCAGATGGTGAGTGCC	79	58.8/ 60.7
ANK1	28–29	CAACGTTCCCGGAGAATG	TTGCCCAGGAGCTTAGTGAC	94	59.0/ 59.3
TMCC2	3–4	CAGAGGGACTACAC-CTACATGAC	CAGTCAGGTCGTTGAGCTG	88	56.6/ 57.1
KLF1	2–3	GGTTGCGGCAAGAGCTAC	GTGCAGGCGTATGGCTTC	83	58.5/ 59.4
AHSP	2–3	TCCGCAGGATTGAAGGAG	CACCACAGTCACCATGTCTTC	90	58.9/ 58.0
GATA1	2–3	TACTACAGGGACGCTGAGGC	AGCCGGCATATGGTGAGC	103	59.3/ 61.1
SDHB	7–8	ATACCGCTGCCACACCATC	TGAA-GCTTTCTTCTCCTTATAGGTTG	118	60.9/ 59.3
MRPL9	4–5	GAGAAGATCCAGACCAAGGC	GGTTCAGCTCCCATTTGAC	100	57.8/ 57.0
CXCL12	1–2	ATGAACGCCAAGGTCGTG	GGCATGGGCATCTGTAGC	97	59.7/ 59.2
DOCK10	52–53	CTGTCGGGCAAGAAGCAC	TCTTCACGTAGGGGAACAGG	88	59.6/ 59.0
ENO2	2–3	CTGAGGGATGGAGACAAACAG	CGCGATGGTGGAGTTGAT	78	58.6/ 59.7
HBG1/2	1–2	GATGCTGGAGGAGAAACCC	CAGGTTGCCAAAGCTGTCA	81	58.6/ 60.0
HBB	1–2	CTGAGGA-GAAGTCTGCCGTTACT	CAAGGGTAGACCACCAGCAG	97	60.1/ 59.6
HBA1/2	1–3	GCTGTCTCCTGCCGACAA	GTCGAAGTGCGGGAAGTAGG	139	60.2/ 61.0
LGALS1	3–4	CTGTCTTTCCCTTCCAGCC	GTATCCATCTGGCAGCTTGAC	89	58.7/ 58.6
H3.3A	4–5	GTGCGAGAAATTGCTCAGGAC	TCAAAAAGGCCAACCAGA-TAGG	104	59.9/ 58.6
ACTB	4	CTGACTGACTACCTCATGAA-GATCCT	CTTAATGTCACGCACGATTTCC	87	60.2/ 60.6

Supplementary Results

Blood count of additional family members

Table S5. Blood count of the father, mother and son 1. The reference values are given in brackets.

		Father (M, 59)	Mother (F, 58)	Son1 (M, 7)	Unit
**White blood cell count**		6.9 (3.5–10)	7.1 (3.5–10)	9.01 (4.5–13.0)	x10^3/μL
**Red blood cell count**		4.90 (4.3 - 6.3)	3.99 (3.7–4.8)	4.68 (3.7–5.8)	x10^6/μL
**Hemoglobin**		15.1 (13.5–17.5)	13.0 (12.0–16.0)	12.8 (10.0–15.0)	g/dL
**Hematocrit**		47.3 (39–49)	41.9 (34–44)	**36.3** (39–49)	%
**Mean corpuscular volume**		95.7 (83–100)	**+105.0** (83–100)	**−77.6** (83–100)	fL
**Mean corpuscular hemoglobin**		30.6 (27–33)	32.6 (27–33)	27.4 (27–33)	pg
**MCHC**		**31.9** (32–35)	**31.0** (32–35)	**35.3** (32–35)	g/dL
**Platelets**		269.0 (150–360)	207.0 (150–360)	**414.0** (150–360)	x10^3/μL
**Lymphocytes**		37.5 (16–45)	42.4 (16–45)	41.0 (16–45)	%
**Neutrophils**		-	-	48.0 (43–75)	%
**RWD_CV**		12.9 (11.0–15.0)	14.2 (11.0–15.0)	13.1 (11.0–15.0)	%
**PDW**		11.5	14.5	-	fL
**Mean platelet volume**		9.3 (7.6–11.2)	11.3 (7.6–11.2)	9.8 (7.6–11.2)	fL
**Platelet-large cell ratio**		20.8	34.5	-	%
**Hemo-globin HPLC**	**HbF**	0.9 (<1)	0.7 (<1)	**+1.6** (<1)	%
	**HbA2**	2.4 (<3.3)	2.6 (<3.3)	3.0 (<3.3)	%
	**Variant hemoglobin**	none	none	none	-

MCHC = mean corpuscular hemoglobin concentration; RWD_CV = variation coefficient of red cell distribution width; PDW= platelet distribution width; bold = values outside reference range.

Pyrosequencing of GATA1 c.886A>C p.(Thr296Pro)

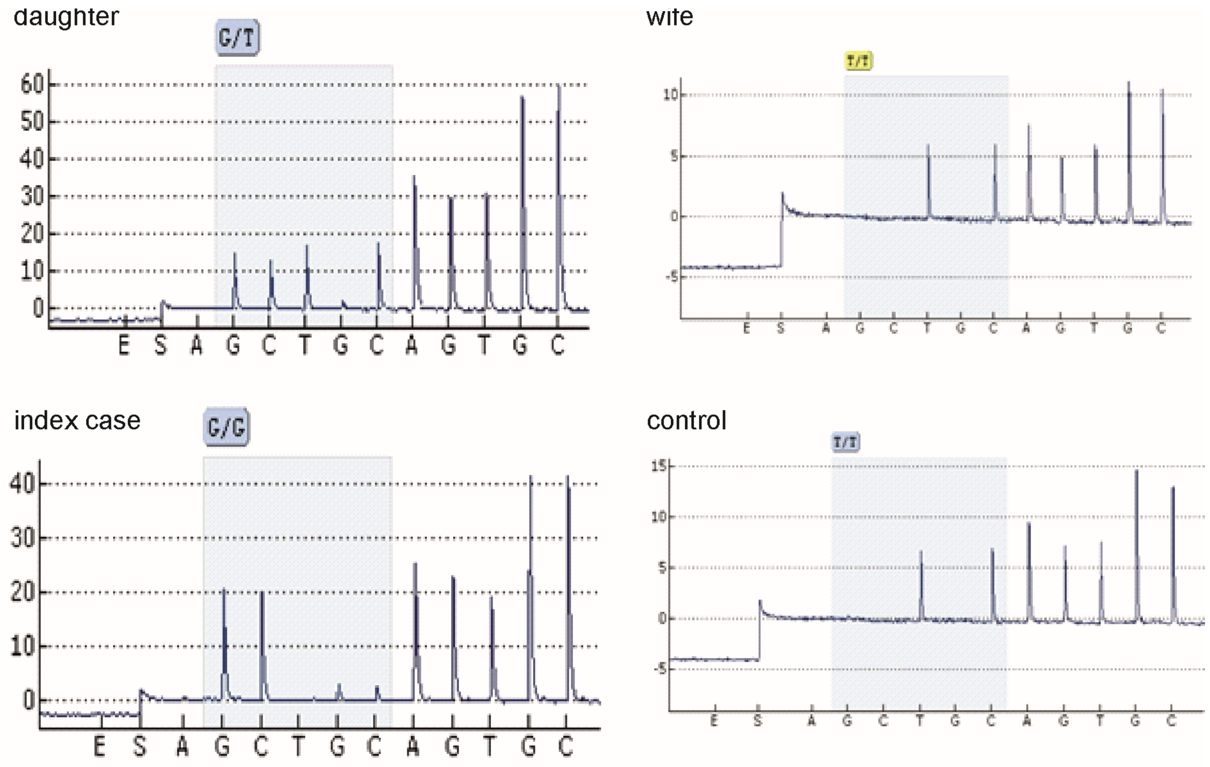

Figure S1. Pyrograms of *GATA1* c.886A>C p.(Thr296Pro) (exon 6, NM_002049.4). The assay uses the reverse strand, therefore, the exchange is T > G here. The daughter is heterozygous for *GATA1* c.886A>C p.(Thr296Pro), the index case is hemizygous, and the control and wife both do not carry the exchange.

Sanger sequencing SLC4A1 c.2210C>T p.(Ala737Val)

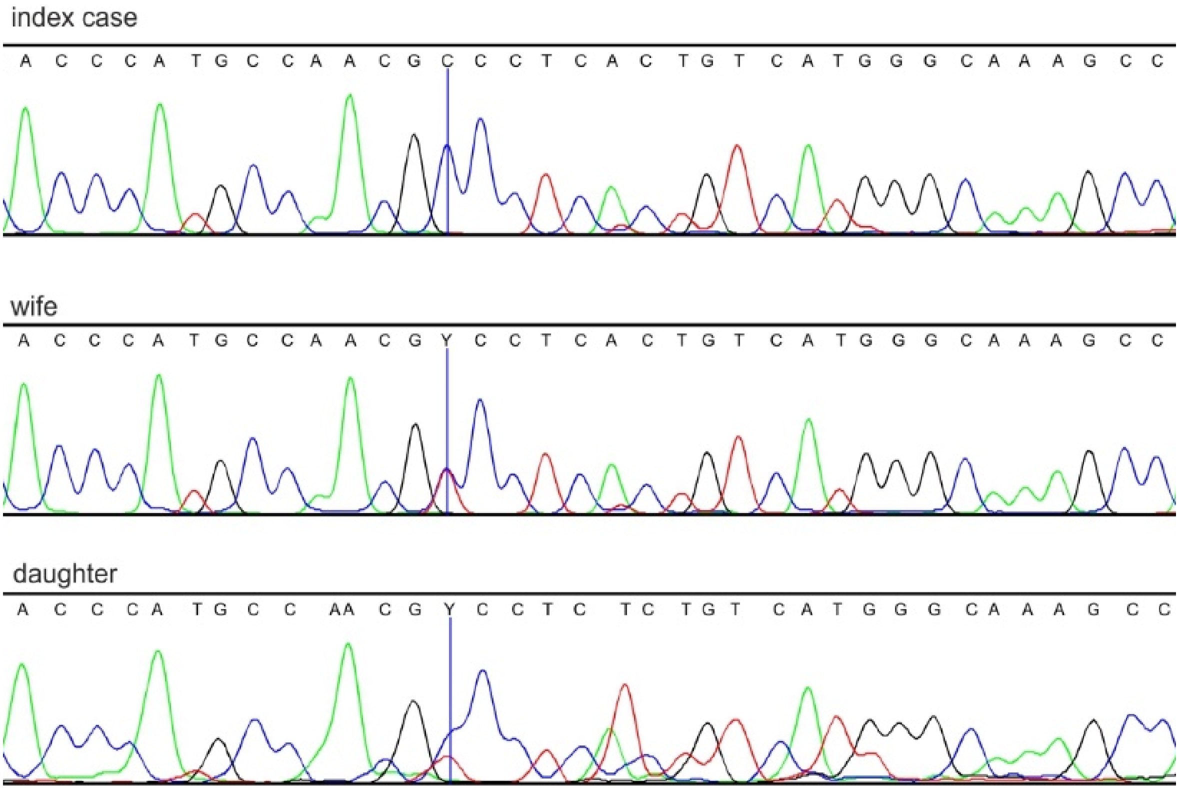

Figure S2. Electropherograms of *SLC4A1* c.2210C>T p.(Ala737Val) (exon 17, NM_000342.4) of the index case, his wife and daughter (blue vertical line = base exchange). The index case (upper panel), his mother, father and son 1 do not carry the variant. His wife (middle panel), son 2, son 3 and daughter (lower panel) are all heterozygous for *SLC4A1* c.2210C>T p.(Ala737Val). Y = T or C.

Surface presentation of major platelet receptors

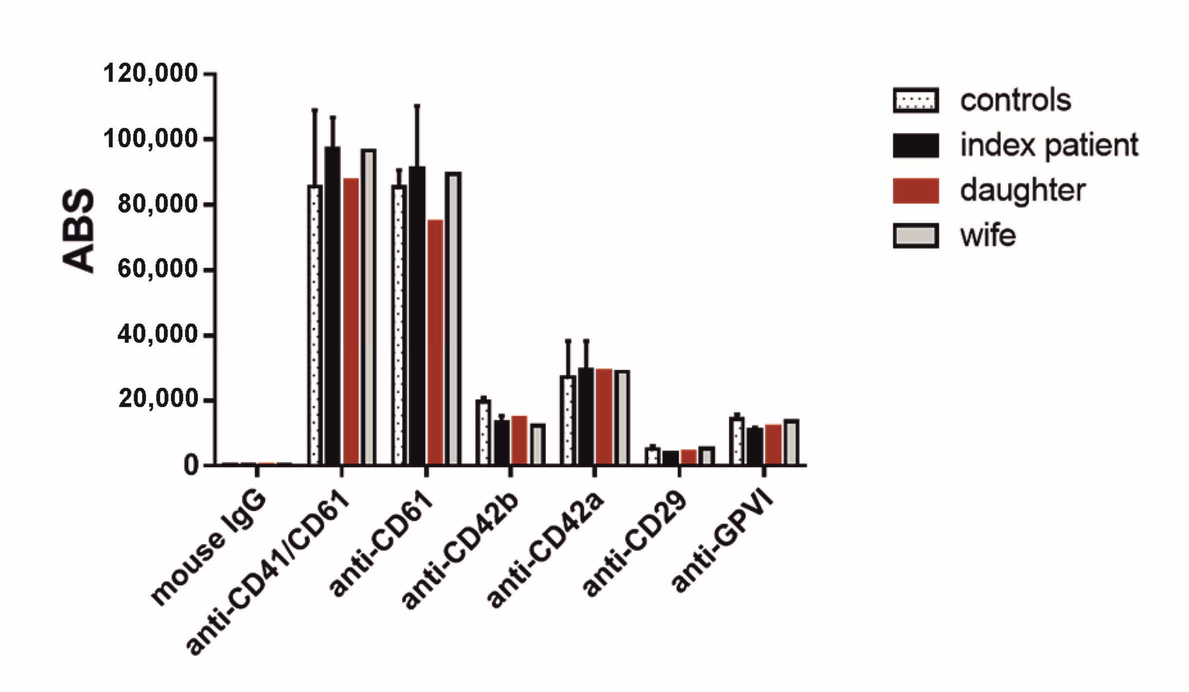

Figure S3. Flow cytometric analysis of major platelet surface receptors from GATA1 index patient, affected daughter, unaffected wife and additional healthy controls. Antigen binding sites (ABS) of anti-GPIIb/IIIa (CD41/CD61), anti-GPIX (CD42a), anti-GPIbα (CD42b), anti-integrin-β1 (CD29) and anti-GPVI antibodies compared to unspecific mouse IgG on platelets in diluted citrated whole blood. Index patient (n = 2), daughter, wife (n = 1), controls (30 different individuals).

mRNA profiling results

Table S6. Transcripts significantly differentially expressed in index case or daughter (overlapping transcripts are highlighted in grey).

Daughter All Significant Transcripts	Transcript ID	Index Case All Significant Transcripts	Transcript ID
ACHE	ENST00000302913	ACHE	ENST00000302913
ACHE	ENST00000411582	AHSP	ENST00000569954
AGPAT1	ENST00000336984	ANK1	ENST00000347528
AHSP	ENST00000569954	BLVRB	ENST00000263368
ANK1	ENST00000347528	BPGM	ENST00000344924
BAG1	ENST00000379704	BTNL3	ENST00000342868
BLVRB	ENST00000263368	CHPT1	ENST00000229266
BPGM	ENST00000344924	CREG1	ENST00000370509
BTNL3	ENST00000342868	CRISP2	ENST00000616725
CRISP2	ENST00000616725	DCAF12	ENST00000361264
CTAG2	ENST00000247306	DDX3Y	ENST00000336079
CTC-490G23.2	ENST00000595748	E2F2	ENST00000361729
CYP26B1	ENST00000412253	EIF1AY	ENST00000361365
DCAF12	ENST00000361264	EIF1AY	ENST00000382772
DMTN	ENST00000265800	FAM210B	ENST00000371384
FAM210B	ENST00000371384	FAM3B	ENST00000398646
FAM46C	ENST00000369448	FAM46C	ENST00000369448
FECH	ENST00000382873	FAM83A	ENST00000276699
GATA1	ENST00000376670	FECH	ENST00000382873
GLRX5	ENST00000331334	FIS1	ENST00000223136
GMPR	ENST00000259727	FUCA1	ENST00000374479
GSPT1	ENST00000563468	GATA1	ENST00000376670
HBBP1	ENST00000433329	GLRX5	ENST00000331334
HBBP1	ENST00000454892	GMPR	ENST00000259727
HBD	ENST00000380299	H2AFJ	ENST00000544848
HBQ1	ENST00000199708	HBBP1	ENST00000433329
HEMGN	ENST00000259456	HBBP1	ENST00000454892
HLA-DQB1	ENST00000434651	HBD	ENST00000380299
HLA-DQB1	ENST00000460185	HBM	ENST00000356815
HLA-F	ENST00000489502	HBQ1	ENST00000199708
IGLC1	ENST00000390321	HIST1H1C	ENST00000343677
IGLC3	ENST00000390325	HLA-DQB1	ENST00000434651
KLF1	ENST00000264834	HLA-F	ENST00000489502
KRT1	ENST00000252244	IGLC3	ENST00000390325
LGALS3	ENST00000254301	KDM5D	ENST00000317961
LILRB2	ENST00000493242	KDM5D	ENST00000382806
LINC01291	ENST00000377469	KDM5D	ENST00000469599
LYL1	ENST00000264824	KLF1	ENST00000264834
MRC2	ENST00000583597	LGALS3	ENST00000254301
NAMPTP1	ENST00000440465	LILRB2	ENST00000493242
NPRL3	ENST00000620134	LINC01291	ENST00000377469
OR2W3	ENST00000360358	LYL1	ENST00000264824
OSBP2	ENST00000535268	MIR4732	ENST00000582320

PDZK1IP1	ENST00000294338	MRC2	ENST00000583597
PPP1R11	ENST00000376763	PDZK1IP1	ENST00000294338
PRDX6	ENST00000470017	PPP1R11	ENST00000376763
PWP2	ENST00000291576	PRKY	ENST00000528056
RP11-734I18.1	ENST00000513211	PRRC2A	ENST00000464890
RPRM	ENST00000325926	RNF11	ENST00000242719
RUNDC3A	ENST00000588564	RPRM	ENST00000325926
SELENBP1	ENST00000368868	RPS4Y1	ENST00000430575
SIAH2	ENST00000312960	RPS4Y1	ENST00000477725
SLC4A1	ENST00000262418	SELENBP1	ENST00000368868
SNCA	ENST00000508895	SIAH2	ENST00000312960
ST6GALNAC4	ENST00000335791	ST6GALNAC4	ENST00000335791
TMCC2	ENST00000329800	TESC	ENST00000335209
TNS1	ENST00000446688	TMCC2	ENST00000329800
TRBV20-1	ENST00000390394	TMEM8C	ENST00000339996
TRIM58	ENST00000366481	TNS1	ENST00000446688
XXyac-YM21GA2.7	ENST00000399186	TRIM58	ENST00000366481
		TSKU	ENST00000333090
		TXLNGY	ENST00000253320
		UBE2M	ENST00000253023
		USP9Y	ENST00000426564
		USP9Y	ENST00000471409
		ZFY	ENST00000155093
